# Mechanistic insights on K_ATP_ channel regulation from cryo-EM structures

**DOI:** 10.1085/jgp.202113046

**Published:** 2022-11-28

**Authors:** Camden M. Driggers, Show-Ling Shyng

**Affiliations:** 1 Department of Chemical Physiology and Biochemistry, School of Medicine, Oregon Health and Science University, Portland, OR

## Abstract

Gated by intracellular ATP and ADP, ATP-sensitive potassium (K_ATP_) channels couple cell energetics with membrane excitability in many cell types, enabling them to control a wide range of physiological processes based on metabolic demands. The K_ATP_ channel is a complex of four potassium channel subunits from the Kir channel family, Kir6.1 or Kir6.2, and four sulfonylurea receptor subunits, SUR1, SUR2A, or SUR2B, from the ATP-binding cassette (ABC) transporter family. Dysfunction of K_ATP_ channels underlies several human diseases. The importance of these channels in human health and disease has made them attractive drug targets. How the channel subunits interact with one another and how the ligands interact with the channel to regulate channel activity have been long-standing questions in the field. In the past 5 yr, a steady stream of high-resolution K_ATP_ channel structures has been published using single-particle cryo-electron microscopy (cryo-EM). Here, we review the advances these structures bring to our understanding of channel regulation by physiological and pharmacological ligands.

## Teaser

Driggers and Shyng present a comprehensive review of recent advances in cryo-EM structures of K_ATP_ channels and the insights from these structures on the mechanisms of channel gating and pharmacology.

## Introduction

Organisms adapt to their energy landscape by eliciting physiological responses in accordance with metabolic demands. K_ATP_ channels are ATP- and ADP-gated potassium channels that regulate membrane potential-dependent cellular activities and thus function as molecular rheostats to maintain metabolic homeostasis. First described in 1983 in cardiac muscles carrying a cyanide-induced outward K^+^ current highly sensitive to inhibition by intracellular ATP (Noma, 1983), related K_ATP_ channels were subsequently found in many other cells including endocrine cells, skeletal muscle, smooth muscle, and neurons. Together, K_ATP_ channels govern a broad range of vital physiological processes, from hormone secretion, skeletal muscle and vascular smooth muscle contraction, cardiac action potential shortening during metabolic stress, to learning and memory. K_ATP_ channel dysfunction underlies several human diseases, including congenital hyperinsulinism (Galcheva et al., 2019; Rosenfeld et al., 2019), neonatal diabetes and DEND (developmental delay, epilepsy, and neonatal diabetes) syndrome (Pipatpolkai et al., 2020), and Cantú syndrome (McClenaghan and Nichols, 2022). In keeping with their importance in human physiology and pathophysiology, K_ATP_ channels are drug targets for a variety of human conditions (Foster and Coetzee, 2016; Kharade et al., 2016). For example, K_ATP_ inhibitors such as sulfonylureas and glinides are widely used for managing type 2 diabetes. The K_ATP_ activator diazoxide is used for treating congenital hyperinsulinism, while pinacidil and cromakalim are activators that have vasodilating effects.

**Figure 1.**
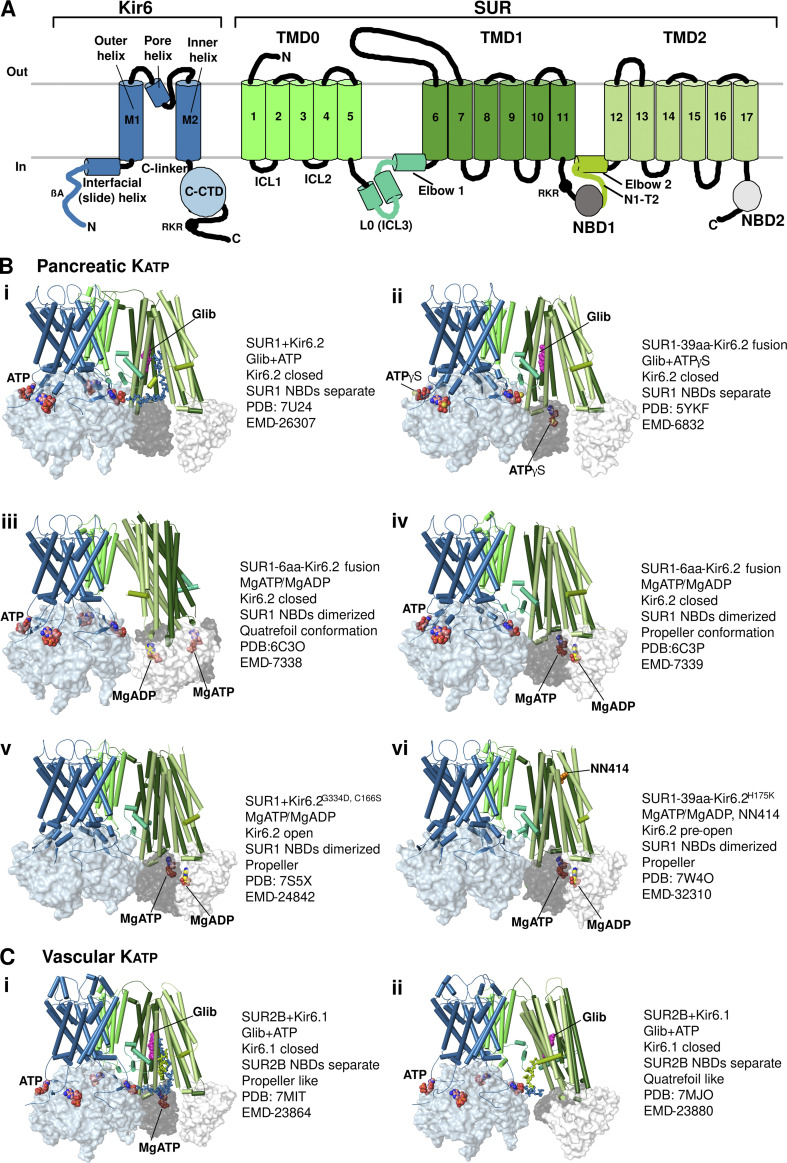
**K**_**ATP**_ **channel structures. (A)** Topology of Kir6 and SUR with major structural domains and elements labeled. TMD, transmembrane domain; C-CTD, C-terminal cytoplasmic domain; ICL, intracellular loop; NBD, nucleotide-binding domain. **(B)** Pancreatic K_ATP_ channels in different liganded states and conformations (only Kir6.2 tetramer and one SUR1 subunit are shown for clarity) as detailed to the right of each structure. **(C)** Vascular K_ATP_ channels bound to Glib and ATP in two different conformations. Note for channels displaying both propeller and quatrefoil conformations, the dominant conformation is shown first.

All K_ATP_ channels are assembled from two proteins, a pore-forming K^+^ channel Kir6.x in the Kir channel family, and a sulfonylurea receptor, SUR, an obligate regulatory subunit in the ABC transporter family (Clement et al., 1997; Inagaki et al., 1996; Inagaki et al., 1995; Fig. 1 A). In humans, these are derived from two alternative pairs of genes. *ABCC8* and *KCNJ11* on chromosome 11 encode SUR1 and Kir6.2; *ABCC9* and *KCNJ8* on chromosome 12 encode SUR2 and Kir6.1, respectively. SUR2 transcripts are alternatively spliced at the last exon which encodes the last C-terminal 42 amino acids to form two major variants, SUR2A and SUR2B. Different Kir6.x and SURx combinations generate different subtypes of K_ATP_ channels with distinct tissue distribution, function, and pharmacology (Tinker et al., 2018). The three best-characterized subtypes are those composed of Kir6.2 and SUR1, Kir6.2 and SUR2A, and Kir6.1 and SUR2B, which are predominantly expressed in pancreatic endocrine cells and neurons, cardiac myocytes, and vascular smooth muscle, respectively, and will be referred to as pancreatic, cardiac, and vascular K_ATP_ channels.

For nearly three decades since the cloning of K_ATP_ channels, a central pursuit of the K_ATP_ field was to understand the structure–function relationship of these complex molecular machineries. Since 2017, a flurry of K_ATP_ structures of wild-type or mutant channels, formed by individual Kir6 and SUR proteins or SUR-Kir6 fusion containing different lengths of linkers, in various ligand conditions have been reported using single-particle cryo-EM. The majority of these structures are of the pancreatic K_ATP_ subtype (Ding et al., 2019; Lee et al., 2017; Li et al., 2017; Martin et al., 2017a; Martin et al., 2019; Martin et al., 2017b; Sung et al., 2022; Sung et al., 2021; Wang et al., 2022; Wu et al., 2018; Zhao and MacKinnon, 2021); only one study has reported structures of the vascular K_ATP_ channel (Sung et al., 2021). These studies have revealed the overall architecture of the channel, the Kir6 tetramer in closed or open conformations, and SUR in inactive NBDs’ separate or activated NBDs-dimerized conformations, as well as the binding sites for pharmacological inhibitors and activators (Fig. 1, B and C). In this review, we discuss mechanistic insights gained from recent cryo-EM structures on physiological and pharmacological regulation of K_ATP_ channels, focusing on the interactions between SUR and Kir6 and their ligands, and the conformational dynamics of the channel relevant to gating. In particular, we highlight new developments in the field since the last review on the topic by Puljung in *JGP* (Puljung, 2018).

## Architecture and assembly domains of K_ATP_ channels

Biochemical and biophysical studies in the 90’s have provided strong evidence that each K_ATP_ channel is a hetero-octamer of Kir6.x and SURx in a 4:4 stoichiometry (Clement et al., 1997; Inagaki et al., 1997; Shyng and Nichols, 1997). Kir6.x contains two transmembrane helices M1 and M2, and cytoplasmic N- and C-terminal domains. SURx are non-transporting members of the ABCC subfamily of ABC transporters (Thomas and Tampé, 2020). Each SUR consists of an N-terminal transmembrane (TM) domain (TMD0) followed by a cytoplasmic linker (L0 or ICL 3) that connects to an ABC core module comprising two TMDs (TMD1 and TMD2) and two cytoplasmic nucleotide-binding domains (NBD1 and NBD2; Fig. 1 A).

**Figure 2.**
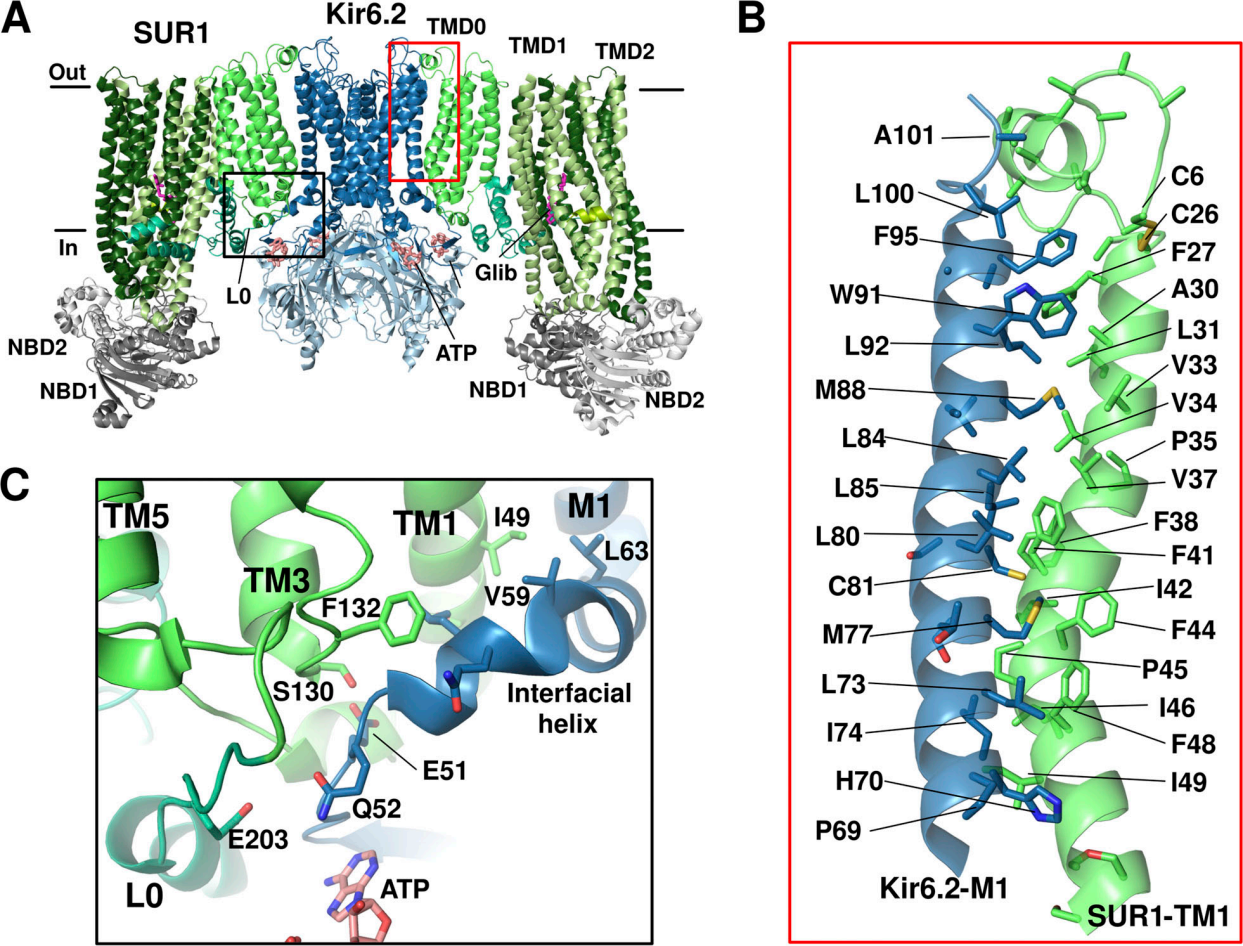
**Assembly domains of pancreatic K**_**ATP**_ **channel. (A)** Overall structure of the pancreatic K_ATP_ channel bound to Glib and ATP (PDB ID 6BAA). Out, extracellular; In, intracellular. Only two SUR1 subunits are shown for clarity. Domain color scheme same as Fig. 1. **(B)** Closeup view of the interface between Kir6.2 and SUR1 at the transmembrane helices (red boxed region in A). **(C)** Closeup view of the detailed molecular interactions between the interfacial helix of Kir6.2, and TMD0 and L0 of SUR1 (black boxed region in A).

The first two pancreatic K_ATP_ structures, one the Kir6.2-GFP/SUR1 channel in the presence of glibenclamide (Glib; Li et al., 2017), and the other the Kir6.2/SUR1 channel in the presence of Glib and ATP (Martin et al., 2017b), both at ∼5–6 Å resolution, confirmed the subunit stoichiometry and revealed the overall 3-D organization of the complex. A tetrameric core of Kir6.2 subunits forms the central transmembrane pore of the channel. A coronal array of four sulfonylurea receptor 1 (SUR1) subunits surrounds the channel core, and each SUR1 is companioned with one Kir6.2 subunit (Fig. 2 A). When viewed perpendicular to the membrane, the complex is shaped like a propeller. In the presence of Glib, the helix bundle crossing (HBC) near the bottom of M2 and the cytoplasmic G-loop gate are tightly constricted (refer to Fig. 9 for the location of these gates), indicating a closed Kir6.2. The ABC module of SUR1 in both structures adopts an inward-facing conformation with the two NBDs well separated. Like other ABC transporters, the TM helices of both TMD1 and TMD2 are domain-swapped such that helices 4 and 5 of each TMD cross over to the other side (see Fig. 1). Based on its structural fold, SUR1 has recently been classified as a type IV ABC transporter, similar to P-glycoprotein, cystic fibrosis transmembrane conductance regulator (CFTR), and multidrug resistance protein 1 (MRP1; Thomas et al., 2020).

These initial structures offer a view of the primary assembly domains between SUR1 and Kir6.2. In particular, the first TM helix in TMD0 of SUR1 makes direct contact with the outer helix M1 of Kir6.2 via a series of hydrophobic interactions (Fig. 2 B), which serve as the primary anchor between the two subunits. Contacts between the intracellular loops of TMD0 and the N-terminal domain of Kir6.2 near the plasma membrane further bind the two subunits together (Fig. 2 C). These domains have been previously implicated in channel assembly and are hotspots for disease-associated mutations that impair channel expression at the cell surface (Martin et al., 2020). Another interface, not resolved in these initial structures but which became clear in subsequent studies, is between the distal Kir6.2 N-terminus (referred to as KNtp) and the central vestibule between the two halves of the SUR1 ABC core (Ding et al., 2019; Martin et al., 2019; Sung et al., 2021; see Fig. 1, B i, C i, and C ii). This interface appears to be rather dynamic and is critical for channel assembly and gating (more discussion in the ATP inhibition and K_ATP_ inhibitors subsections).

## Structural insights on K_ATP_ channel regulation by physiological ligands

The physiological activity of K_ATP_ channels is a balance between two opposing effects of intracellular ATP and ADP (Nichols, 2006). ATP and, to a lesser extent, ADP inhibit channel activity in an Mg^2+^-independent manner by binding to inhibitory sites on Kir6, while in the presence of Mg^2+^, ADP, and, to a lesser extent, ATP also stimulate channel activity by binding to stimulatory sites on SUR. As intracellular ATP/ADP ratios increase, the inhibitory effects of the nucleotides prevail, leading to an overall suppression of channel activity. Conversely, when intracellular ATP/ADP ratios decrease, the stimulatory effects of the nucleotides push the equilibrium toward channel opening. Although all K_ATP_ isoforms are regulated by intracellular ATP and ADP, their nucleotide sensitivities differ (Foster and Coetzee, 2016; Fujita and Kurachi, 2000), likely tailored to their metabolic environment and physiological function. For example, the pancreatic K_ATP_ is sensitive to the fluctuations of intracellular ATP and ADP that occur under physiological glucose concentrations to regulate insulin secretion (Ashcroft, 2006). By contrast, cardiac K_ATP_ channels open only under ischemic or hypoxic conditions to shorten cardiac potentials for cardioprotection (Foster and Coetzee, 2016; Nichols et al., 2013). Vascular K_ATP_ channels that control vascular tone are highly dependent on Mg-nucleotides for activation and are much less sensitive to ATP inhibition (Foster and Coetzee, 2016; Nichols et al., 2013; Tinker et al., 2018).

Early studies using a C-terminally truncated Kir6.2 lacking an endoplasmic reticulum (ER) retention signal (the RKR motif) and thus able to reach the cell surface without SUR (Kir6.2ΔC channels) showed that such channels are still inhibited by ATP and ADP (Tucker et al., 1997), but are not stimulated by MgATP/ADP (Gribble et al., 1998). This led to the conclusion that the nucleotide inhibitory sites reside in the Kir6 subunit, while the SUR subunit harbors the nucleotide stimulatory sites. Another key player for K_ATP_ function is membrane phospholipids, in particular, phosphatidylinositol-4,5-bisphosphate (PIP_2_). In vitro application of PIP_2_ or overexpression of a PI-5-kinase that increases cellular PIP_2_ production increase channel opening probability (*P*_*o*_) and render the channel less sensitive to ATP inhibition (Baukrowitz et al., 1998; Shyng et al., 2000a; Shyng and Nichols, 1998). The effect of PIP_2_ on K_ATP_ channels has been attributed to a binding site in Kir6, akin to the sites revealed in other Kir channels, such as Kir2 and Kir3. However, although Kir6.2 alone is sensitive to ATP/ADP inhibition and PIP_2_ stimulation, SUR1 enhances sensitivity to both ligands (Enkvetchakul et al., 2000). Recent cryo-EM structures have begun to shed light on how SUR and Kir6 interact to confer the gating behavior of the K_ATP_ channel complex.

### ATP inhibition

**Figure 3.**
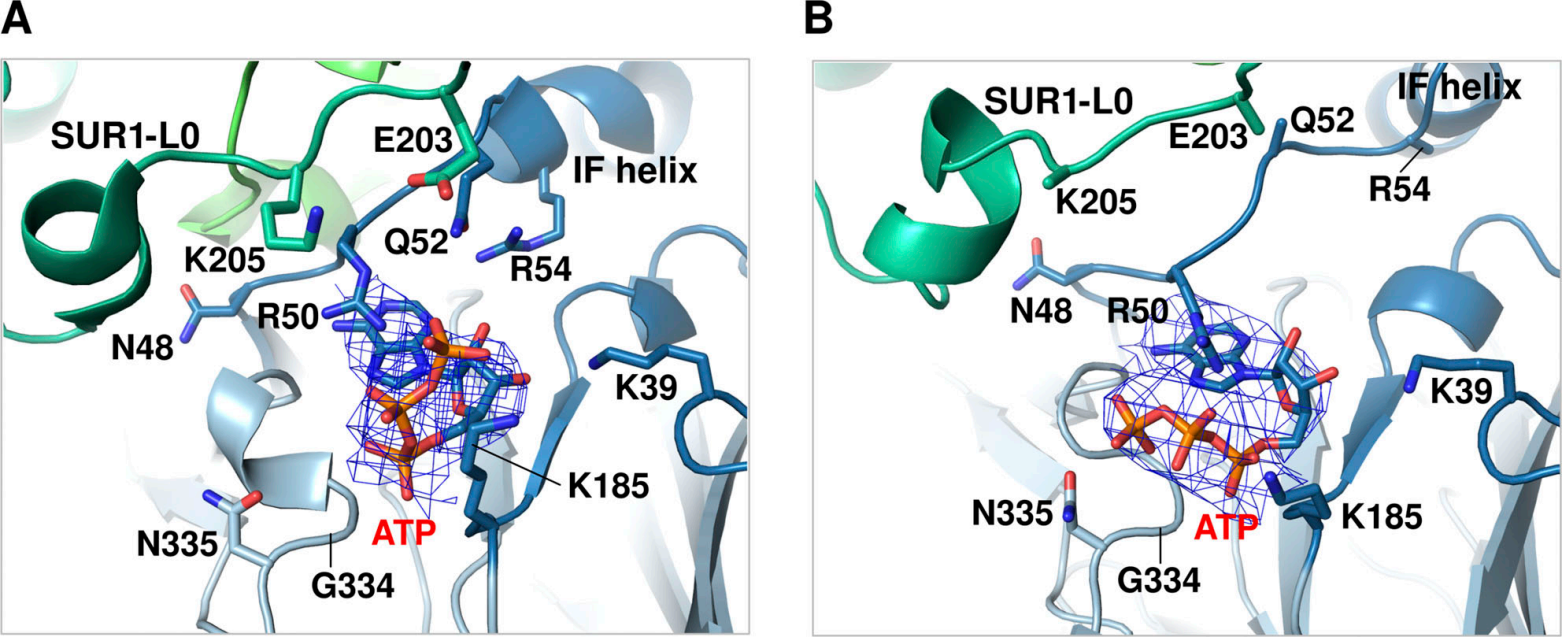
**ATP binding pocket showing two alternative ATP poses. (A)** ATP binding site model from the cryo-EM structure of the ATP- and Repa-bound K_ATP_ channel with Kir6.2-CTD in the up conformation (PDB ID 7TYS). Note the sidechain of K205 from SUR1-L0 is oriented toward the bound ATP. The ATP density (blue mesh; EMD-26193) is fitted with a pose in which the γ-phosphate tail is pointed up toward R50. **(B)** ATP binding site model from the cryo-EM structure of human K_ATP_ determined in the presence of MgATP in propeller form (PDB ID 6C3P). The ATP density (blue mesh; EMD-7339) is fitted with a pose in which the γ-phosphate tail is facing Kir6.2-N335. Note that in B, several sidechains were not resolved in the cryo-EM maps and therefore were not modeled, including sidechains of Kir6.2 residues Q52 and R54, and SUR1 residues E203 and K205 (Lee et al., 2017).

Rapid and reversible inhibition by ATP is a unique property that sets K_ATP_ channels apart from other Kir channels (Hibino et al., 2010). Structures of pancreatic K_ATP_ where Kir6.2 is bound to ATP (PDB IDs 6BAA, 6C3O), ATPγS (5YKF, 5YW8), or ADP (5YWC, 7W4P) are now available (only PDB models from cryo-EM maps <5 Å are mentioned here). In these structures, the nucleotide-binding site is directly below the inner membrane leaflet at the interface of two adjacent Kir6.2 subunits. Nucleotide binding is primarily coordinated by residues from the N-terminal peptide immediately before the “interfacial helix” (aka “slide helix”) of one subunit and the C-terminal cytoplasmic domain of the neighboring subunit (Fig. 3). Of note, the cryo-EM density of ATP has been modeled in two alternative poses. In one, the γ-phosphate is oriented toward R50 of Kir6.2 (Fig. 3 A; Martin et al., 2017a; Wu et al., 2018), and in the other, the ATP’s γ-phosphate faces N335 (Fig. 3 B; Lee et al., 2017). Both poses are consistent with previous functional data implicating R50 and N335 in ATP binding (Drain et al., 1998; John et al., 2003; Trapp et al., 1998). Likely the density represents an ensemble of the two possible γ-phosphate poses. Sequence comparison between Kir6 and other Kir channels reveals that G334 in Kir6 is replaced by larger amino acids at the equivalent position, such as histidine in Kir2 and Kir3. Substitution of glycine by a larger amino acid would create steric hindrance and prevent ATP binding. This may explain, at least in part, why K_ATP_ channels are sensitive to ATP regulation while other Kir channels are not. Indeed, a G334D mutation has been recently exploited to obtain open channel structure by preventing ATP binding at the inhibitory site in Kir6.2, even in the presence of MgATP/ADP that induces dimerization of SUR1 NBDs (Zhao and MacKinnon, 2021; see below).

In addition to residues from Kir6.2, K205 of SUR1 in the L0 loop, immediately following TMD0, has its side chain pointed toward the β- and γ-phosphates of ATP in the cryo-EM structures (Ding et al., 2019; Sung et al., 2022; Fig. 3). Mutation of K205 to alanine or glutamate decreased the IC_50_ for ATP inhibition (Ding et al., 2019; Pratt et al., 2012). Further evidence that SUR1-K205 directly contributes to ATP binding came from a study by Usher et al. (2020). Here, ATP binding affinity assessed by fluorescence resonance energy transfer (FRET) between a fluorescent ATP analog and a fluorescent unnatural amino acid (3-[6-acetylnaphthalen-2-ylamino]−2-aminopropanoic acid; ANAP) placed at Kir6.2 amino acid position 311 showed that SUR1-K205A and K205E mutations reduce ATP binding by ∼5- and 10-fold, respectively. These observations provide a mechanism by which SUR1 enhances the ATP sensitivity of Kir6.2. However, previous studies found that coexpressing Kir6.2ΔC with SUR1-TMD0 plus increasing lengths of L0 that include K205 yielded channels that were still much less sensitive to ATP inhibition (Babenko and Bryan, 2003), indicating SUR1 regions outside TMD0-L0 are at play.

**Figure 4.**
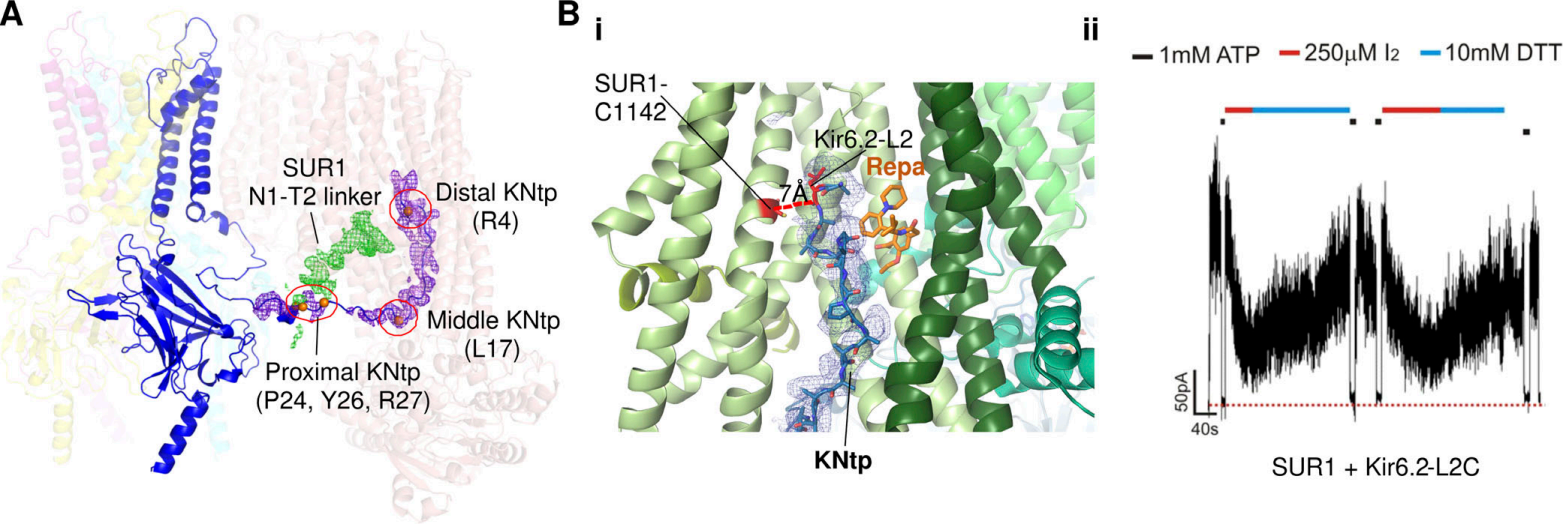
**Interface between KNtp and SUR-ABC core. (A)** Cryo-EM structure of Kir6.2 tetramer plus one SUR1 bound to Repa and ATP in Kir6.2-CTD up conformation is shown with KNtp modeled for one Kir6.2 subunit (blue; PDB ID 7TYS). The cryo-EM density of Kir6.2-KNtp is shown as a purple mesh and that of SUR1 N1–T2 linker as a green mesh (EMD-26193, filtered to 5 Å and contoured to 0.7 σ). Residues along KNtp making discernable contacts with SUR1 are shown as orange spheres and circled in red. **(B)** Crosslinking of distal KNtp with SUR1 reduces channel activity. **(B i)** Structure of the SUR1 subunit (green helices) bound to Repa (orange carbons) showing the proximity of KNtp (blue carbon sticks in blue cryo-EM density) to the bound Repa and the relative position of Kir6.2-L2 to SUR1-C1142 (orange; PDB ID 6PZ9; EMD-20528, filtered to 6 Å and contoured to 12 σ). **(B ii)** Inside-out patch-clamp recording showing that exposure of SUR1+Kir6.2-L2C channels to the oxidizing agent iodine (I_2_) reduced channel activity, which was reversed by exposure to the reducing agent dithiothreitol (DTT), indicating immobilizing KNtp at the interface inhibits channel activity. The red dashed line indicates baseline zero currents (adapted from Fig. 5 A in Martin et al., 2019).

As discussed in detail in the Pharmacological modulators of K_ATP_ channels section, cryo-EM structures of K_ATP_ channels formed by separate SUR1 and Kir6.2 proteins bound to pharmacological inhibitors and/or ATP have uncovered the density of Kir6.2 N-terminal peptide (approximately the first 30 amino acids; referred to as KNtp) in the vestibule between the two halves of the SUR1 ABC core module (Martin et al., 2019; Sung et al., 2022; Fig. 4 A). By contrast, no KNtp density was seen when no inhibitory ligands were included (Martin et al., 2019), suggesting inhibitory ligands promote interactions at the interface between KNtp and SUR1. The KNtp has long been known to have a critical role in regulating K_ATP_ activity. Deletion of KNtp increases channel *P*_*o*_ and allosterically reduces channel sensitivity to ATP inhibition (Babenko et al., 1999; Koster et al., 1999b; Reimann et al., 1999). Cryo-EM structure-guided crosslinking between an engineered cysteine at amino acid position 2 of Kir6.2 (Leu to Cys) and a SUR1 endogenous cysteine (C1142) in TM14 lining the ABC core central cavity inhibits channel activity in the absence of ATP (Martin et al., 2019; Fig. 4 B), evidence that this interface stabilizes channel closure. Taken together, a picture emerges wherein SUR1 enhances Kir6.2 sensitivity to ATP inhibition by (1) directly participating in ATP binding and (2) stabilizing Kir6.2 N terminus in the SUR1 ABC core central cavity, which promotes channel closure. Conceptually, this means that the apparent affinity of ATP inhibition is determined not only by the “binding affinity” set by ATP interacting residues but also by interactions that stabilize Kir6.2 in a closed state. It has been shown previously that the mutation pair SUR1-E203K and Kir6.2-Q52E increased the apparent affinity for ATP inhibition by >100-fold, and crosslinking of the two residues by engineered cysteines locked the channel in a closed state even in the absence of ATP (Pratt et al., 2012). In cryo-EM structures of K_ATP_ bound to ATP, SUR1-E203 and Kir6.2-Q52 are in close proximity. The study illustrates how manipulating SUR1-Kir6.2 interactions to stabilize the closed state has profound effects on channel ATP sensitivity.

### Gating by phospholipids

There is extensive experimental evidence that membrane phospholipids are involved in the physiological activity of K_ATP_ channels (Nichols, 2006). In inside-out patch-clamp recording experiments in the absence of ATP and ADP, bath application of phosphoinositides, in particular PI4,5P_2_, increases channel activity (i.e., open probability) while neomycin and polylysine, which sequester negatively charged headgroups of phospholipids, reduce channel activity (Baukrowitz et al., 1998; Shyng and Nichols, 1998). Overproduction of PI4,5P_2_ in cells also increases K_ATP_ channel activity (Lin et al., 2005; Shyng et al., 2000a). Since PI4,5P_2_ is the most abundant phosphoinositide present in the plasma membranes and it similarly activates other Kir channels including Kir2 and Kir3, it has been assumed as an essential endogenous activating ligand of K_ATP_ channels. However, a recent study showed that purified human K_ATP_ channels reconstituted in lipid bilayers composed of DOPE (dioleoylphosphatidylethanolamine), POPC (1-palmitoyl-2-oleoyl-sn-glycero-3-phosphocholine), and POPS (1-palmitoyl-2-oleoyl-sn-glycero-3-phospho-L-serine) but lacking PI4,5P_2_ undergo spontaneous openings that are inhibited by ATP, arguing against an absolute requirement of PIP_2_ for K_ATP_ activity (Zhao and MacKinnon, 2021). Of note, in contrast to crystal or cryo-EM structures of Kir2 and Kir3 determined in the presence of diC8-PIP_2_ showing a clear density of the lipid in its binding pocket, none of the cryo-EM K_ATP_ structures imaged in the presence of diC8-PIP_2_ showed definitive PIP_2_ density in the predicted PIP_2_ binding pocket (Lee et al., 2017; Wang et al., 2022; Wu et al., 2018). This is the case even when Kir6.2 H175 was mutated to lysine to match the corresponding residue in Kir2 and Kir3, which presumably increases PIP_2_ binding affinity for Kir6.2 (Wang et al., 2022). It remains a possibility that even with this mutation, K_ATP_ channels still have lower affinity and/or selectivity for PIP_2_ (Rohács et al., 1999; Rohács et al., 2003). Thus, whether PIP_2_ physiologically binds at the predicted pocket and determines the activity of K_ATP_ channels remain unresolved.

**Figure 5.**
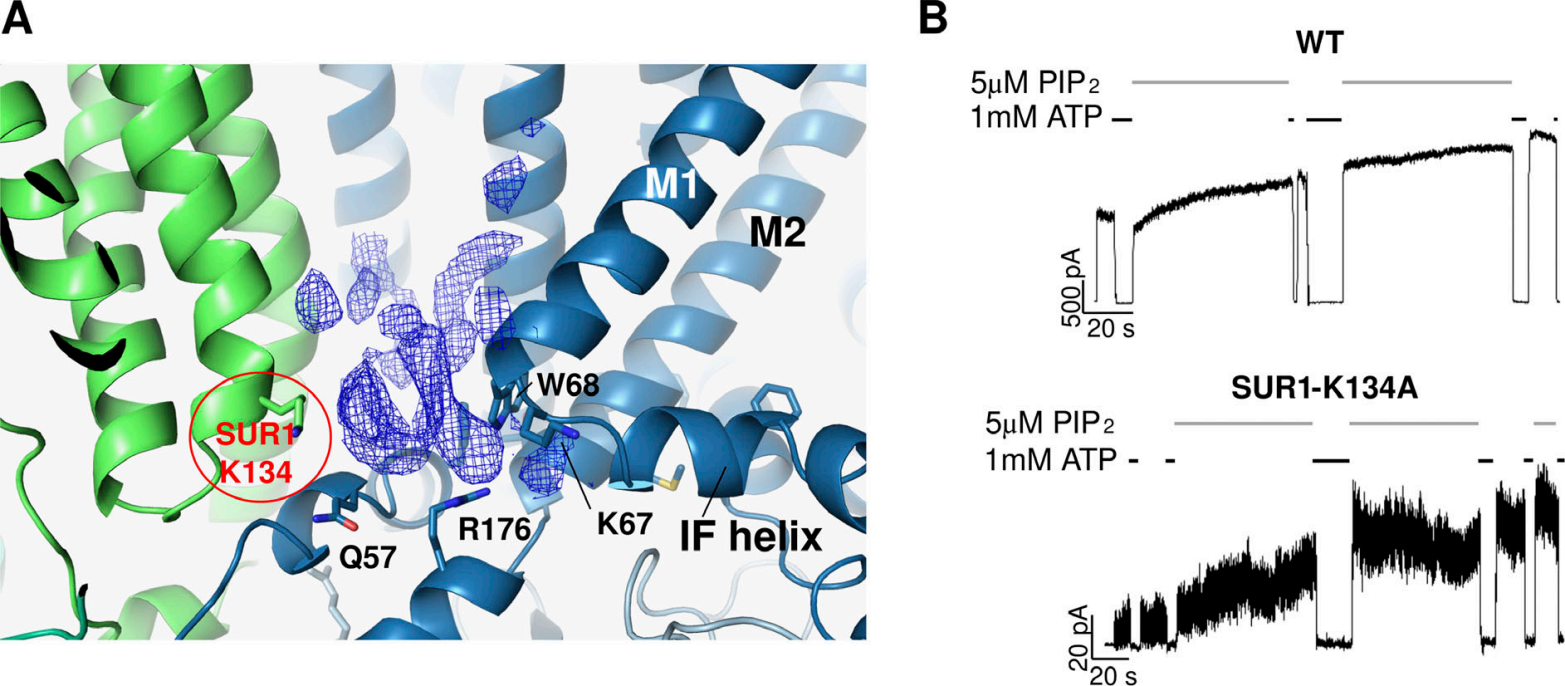
**SUR1 contributes to PIP**_**2**_ **regulation of K**_**ATP**_ **channels. (A)** Cryo-EM structure model of the Kir6.2/SUR1 channel bound to Repa and ATP in the Kir6.2-CTD up conformation (PDB ID 7TYS) showing that SUR1-K134 (red label) is adjacent to a potential lipid density (EMD-26193) in the proposed PIP_2_ binding pocket of Kir6.2. **(B)** Inside-out patch-clamp recordings comparing the response of WT and SUR1-K134A mutant channels to PIP_2_ (adapted from Fig. 5 in Sung et al., 2022). Top: The WT channel showed a characteristic opening response to PIP_2_ exposure, and closure upon exposure to ATP. Bottom: The SUR1-K134A mutant showed significantly lower initial activity, which was greatly increased upon PIP_2_ exposure (5.76 ± 2.11-fold increase versus 1.30 ± 0.23-fold increase for WT channels, *n* = 4–6), suggesting SUR1-K134 contributes to PIP_2_ sensing and gating.

Unlike Kir2 and Kir3, Kir6.x forms a complex with SURx to make up the K_ATP_ channel. It has long been recognized that while Kir6.x alone is sensitive to PIP_2_ regulation, SURx markedly increases Kir6.x sensitivity to PIP_2_, as reflected by a >10-fold higher nucleotide-independent open probability of the Kir6.2/SUR1 channel compared with the Kir6.2 channel alone (Enkvetchakul et al., 2000). Early work has shown that TMD0 from SUR1 is sufficient to confer the high open probability of Kir6.2/SUR1 channels (Babenko and Bryan, 2003; Chan et al., 2003); however, the underlying structural mechanism has remained unclear. Despite the controversy on PIP_2_ and K_ATP_ channel interactions discussed above, a recent study of K_ATP_ cryo-EM structures did provide evidence that SUR1 enhances K_ATP_ channel sensitivity to phospholipids/PIP_2_ by contributing to phospholipid binding (Sung et al., 2022). In a structure of the Kir6.2/SUR1 channel bound to repaglinide (Repa) and ATP, a lysine residue located in ICL2 of the SUR1–TMD0 domain (K134) has its side chain directed toward the head group of the lipid density in the proposed Kir6.2 PIP_2_ binding pocket (Fig. 5 A). Mutating this lysine to alanine reduced channel *P*_*o*_, which was recovered by adding exogenous PIP_2_ (Fig. 5 B). These findings support the notion that SUR1–K134 is involved in phospholipid/PIP_2_ binding, thus providing a structural mechanism of how SUR1, specifically TMD0 of SUR1, enhances Kir6.2 sensitivity to phospholipids/PIP_2_.

### Mechanism of ATP and PIP_2_ antagonism

ATP and PIP_2_ functionally compete to stabilize channels in the closed and open states, respectively. Alanine scanning mutagenesis of positively charged residues in the N- and C-terminal cytoplasmic domains of Kir6.2 has identified mutations that reduce channel response to ATP, PIP_2_, or both (Cukras et al., 2002; Shyng et al., 2000b). The finding that some mutations reduce channel response to both ligands led to a hypothesis that these mutated residues may be involved in the binding of both ligands. Recently, molecular dynamics studies found that Kir6.2 residues R54 and K39 interact with both ATP and PIP_2_ (Pipatpolkai et al., 2022; Sung et al., 2022). Mutation of both R54 and K39 to alanine has been shown to reduce ATP as well as PIP_2_ sensitivities (Cukras et al., 2002). Thus, there is now converging structural and functional evidence that ATP and PIP_2_ functionally compete by sharing overlapping but non-identical residues. In cryo-EM structures of open K_ATP_ channels, the ATP binding pocket is remodeled such that ATP/ADP no longer binds (Wang et al., 2022; Zhao and MacKinnon, 2021), implying that the inhibitory binding of ATP/ADP and the binding of phospholipids/PIP_2_ are mutually exclusive. Speculation is that switching of R54 and K39 between ATP and PIP_2_ binding may provide a pathway to exclusive binding of one of the ligands by facilitating dissociation of the other.

### MgADP stimulation

Mg–nucleotide stimulation in K_ATP_ channels is mediated by the SUR subunit. Like many ABC exporters from eukaryotic organisms, SUR contains two asymmetric NBDs (Stockner et al., 2020): NBD1 binds MgATP/ADP but cannot hydrolyze ATP (the degenerate site) and NBD2, which binds MgADP with higher affinity than MgATP, retains ATPase activity (the consensus site), although the ATPase activity is quite low (0.02–0.03/s or >50 s for each ATP hydrolysis cycle; de Wet et al., 2007; Lee et al., 2017). It has been widely assumed that, as in other ABC transporters, MgATP/ADP induces NBD dimerization. Using a human SUR1-6aa-Kir6.2 fusion construct, Lee et al. (2017) published the first K_ATP_ structure in which the NBD1 and NBD2 of SUR1 are respectively bound to MgATP and MgADP and dimerized (Lee et al., 2017). The NBD-dimerized SUR1 does not show an outward-facing conformation as in typical ABC exporters, consistent with SUR1 not having transport activity (Aittoniemi et al., 2009). Although MgADP/MgATP-induced NBD dimerization at SUR1 is expected to open K_ATP_ channels, Kir6.2 is still bound to ATP and closed, likely because the high concentrations of MgATP included in the cryo-EM sample saturated the inhibitory ATP binding site at Kir6.2 to override the activating effect of SUR1 (Lee et al., 2017).

**Figure 6.**
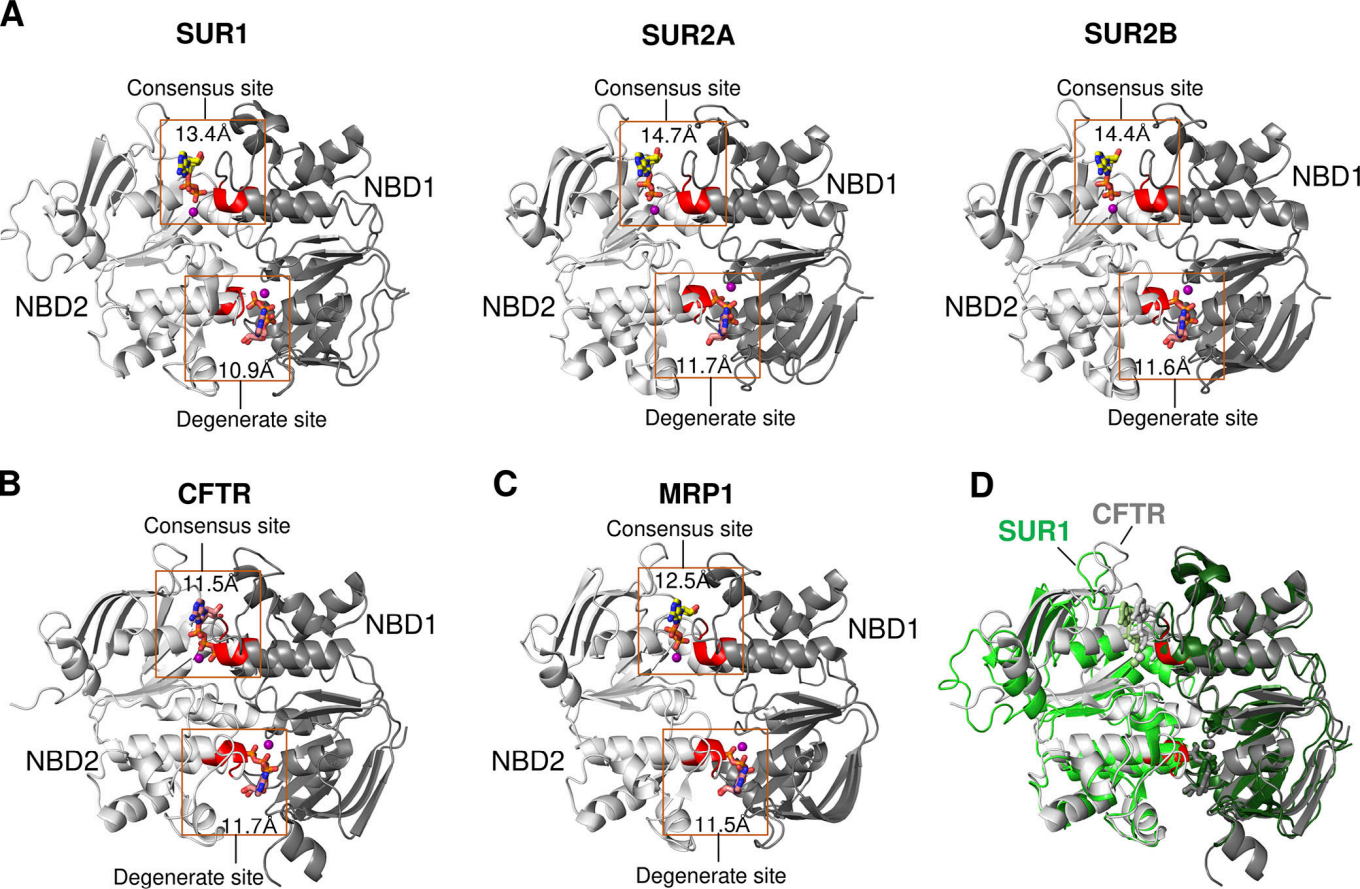
**Comparison of the consensus and degenerate sites in Mg-nucleotide bound, NBD-dimerized cryo-EM structures of SURs and related ABCC transporters. (A)** Dimerized NBD structures of SUR1 (PDB ID 7S5V), SUR2A (PDB ID 7VLU), and SUR2B (PDB ID 7VLT) showing the consensus site bound to MgADP (yellow carbons) is more open than the degenerate site bound to MgATP (salmon carbons). Distance (Å) shown in the box is between the Cα-atoms of Walker A lysine and the second glycine of the signature sequence. **(B and C)** NBD-dimerized structures of the homologous ABCC subfamily proteins CFTR (PDB ID 6MSM; the E1371Q mutant human CFTR with MgATP bound to both consensus and degenerate sites) and MRP1 (PDB ID 6UY0) showing both the consensus and degenerate sites are tightly closed. **(D)** Overlay of the human CFTR structure shown in B (grey) and the SUR1 structure shown in A (green).

Several K_ATP_ or SUR structures in which the SUR NBDs are dimerized have now been reported by incubating purified K_ATP_ channels/SUR with MgATP (ATP at 8–10 mM; Lee et al., 2017; Zhao and MacKinnon, 2021) or MgADP (ADP ranging from 0.5 to 8 mM; Ding et al., 2022; Wang et al., 2022; Wu et al., 2018). In the case where MgATP was used, the degenerate site was occupied by MgATP but the consensus site was occupied by MgADP, which could result from MgATP hydrolysis or from contaminating MgADP present in MgATP. In the case where MgADP was used, an initial report modeled the nucleotide density at both sites as MgADP (Wu et al., 2018); however, two subsequent studies found the nucleotide at the degenerate site to be MgATP, which was attributed to contaminating ATP in ADP, and that at the consensus site to be MgADP (Wang et al., 2022; Wu et al., 2018). Regardless of sample conditions or interpretation of the bound nucleotides, the dimerized NBD structures of SURs are characteristic of ABC transporters showing canonical MgATP/ADP binding sites formed by the A loop, Walker A, and Walker B motifs from one NBD and the signature sequence from the other NBD arranged in a “head-to-tail” fashion (Fig. 6 A). Quite strikingly, in all the structures, the signature sequence from NBD1 at the consensus site is disengaged from the bound MgADP such that the consensus site is more open than the degenerate site (Fig. 6 A). The dimerized NBD structures of SURs revealed by cryo-EM suggest the Mg–nucleotide at the consensus site will be able to exchange with Mg–nucleotides in the solution, consistent with many studies indicating that Mg–nucleotide regulation of K_ATP_ channels does not involve ATP hydrolysis (Choi et al., 2008; Proks et al., 2010; Sikimic et al., 2018).

Compared with SUR, two other ABCC proteins that also have functionally asymmetric NBDs require ATP hydrolysis to function: CFTR, a chloride channel, and MRP1, a transporter. Interestingly, in human CFTR (E1371Q mutant; PDB ID 6MSM; Zhang et al., 2017) and bovine MRP1 (PDB ID 6UY0) NBD-dimerized structures (Wang et al., 2020), both consensus and degenerate sites are tightly closed (Fig. 6, B–D). These structural and functional differences provide insights into how SUR can rapidly and effectively sense intracellular MgADP concentration change at the consensus site to regulate channel activity. It remains unclear what the functional role of ATP hydrolysis is, if any, in K_ATP_ regulation.

Following SUR NBD dimerization by MgADP, what is the structural crosstalk between SUR and Kir6 that leads to channel stimulation even in the presence of inhibitory ATP? In the first study showing K_ATP_ channel structures with dimerized SUR1 NBDs, two distinct conformations are reported: a minor class (∼23% of classified particles), similar to the inhibitor-bound propeller conformation, and a dominant class (∼77% of classified particles) that resembles a quatrefoil wherein the SUR1 subunits are rotated clockwise by ∼63° around the central axis of the Kir6.2 tetramer (viewed from the extracellular side). In the quatrefoil conformation, a new interface between NBD2 and the cytoplasmic domain of the Kir6.2 domain is observed (Lee et al., 2017), suggesting a potential crosstalk pathway. However, whether this interface is involved in SUR1 activation of Kir6.2 has not been tested. In contrast to the dramatic movement of the SUR1 ABC module seen in the quatrefoil conformation, more subtle movements of TMD0 and L0 have been proposed to account for the MgADP stimulation effect by comparing the structure of a SUR1-39aa-Kir6.2^H175K^ channel in NBDs dimerized and pre-open state with that of a SUR1-39aa-Kir6.2 channel bound to the inhibitor Repa and ATP, both in propeller conformations (Wang et al., 2022). Here, an outward movement of the L0 and the inner half of TMD0 pulls the ATP coordinating residue K205 in L0 of SUR1 away from the ATP binding site in Kir6.2, which would reduce ATP binding and inhibition. In addition to this mechanism, an open K_ATP_ structure of an NBD-dimerized SUR1 and Kir6.2^G344D, C166S^ shows no Kir6.2 Ntp cryo-EM density in the SUR1 ABC core cavity (Zhao and MacKinnon, 2021). Release of the KNtp from SUR1 would also increase channel open probability, which would indirectly reduce ATP sensitivity (Enkvetchakul et al., 2000).

## Pharmacological modulators of K_ATP_ channels

### K_ATP_ inhibitors

**Figure 7.**
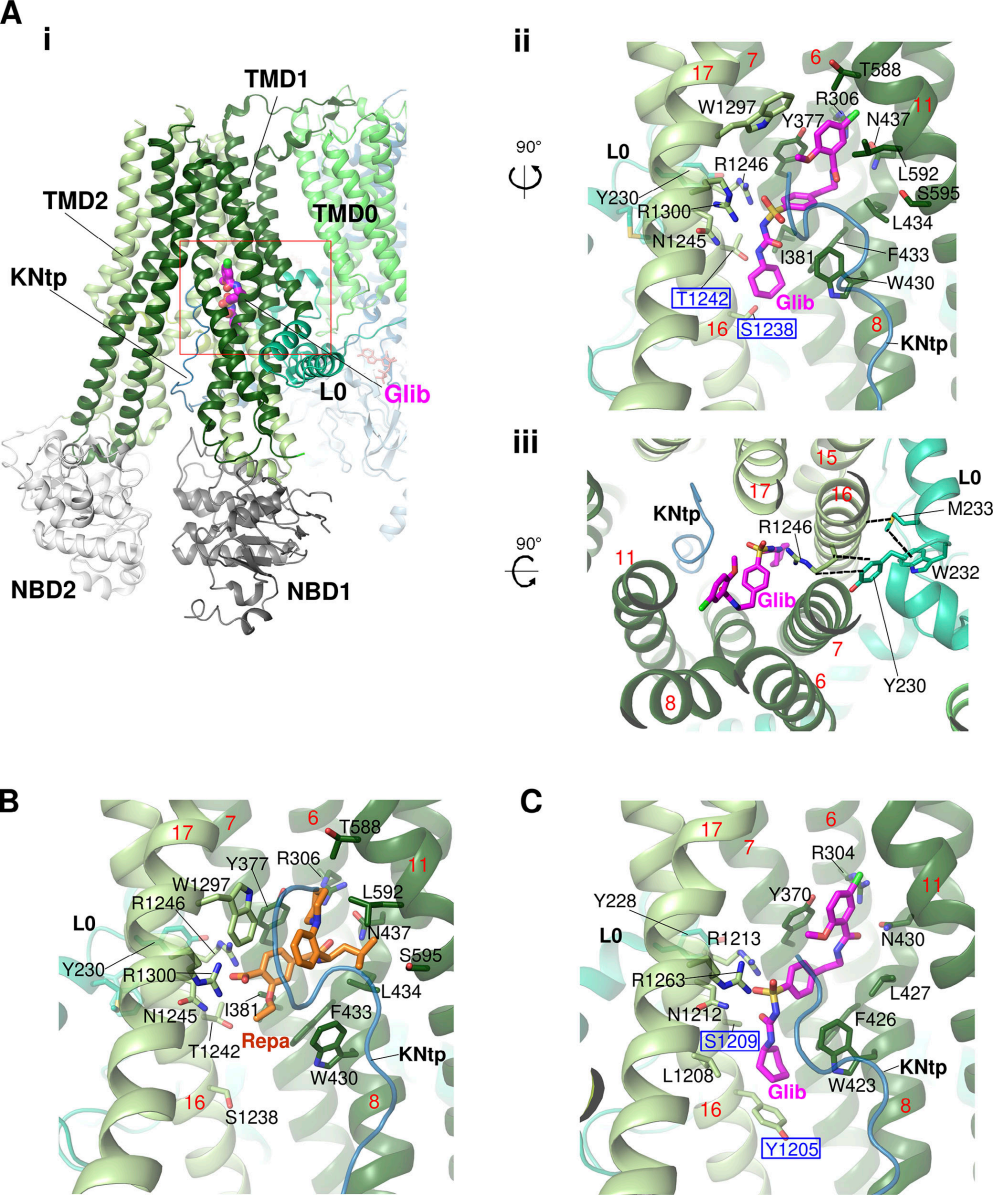
**Binding sites of pharmacological inhibitors. (A)** Glibenclamide (Glib) binding pocket in the Kir6.2/SUR1 (pancreatic) K_ATP_ channel (PDB ID 7U24). **(A i)** Overview of the Glib binding pocket from the side. **(A ii)** Closeup side view of the Glib binding pocket with surrounding residues labeled. The two residues that are different between SUR1 and SUR2B (see C) are labeled in blue font with a blue outline. The red numbers indicate TM helix numbers in this and subsequent panels. **(A iii)** Glib binding pocket viewed from the top, highlighting the interactions between L0 residues (cyan carbons) and TMD1/2 helices lining the pocket (indicated by dashed lines). **(B)** The same pocket as shown in A accommodates another inhibitor repaglinide (Repa) but with a distinct set of interacting residues (compare to A ii; PDB ID 7TYS). **(C)** Closeup side view of the Glib binding pocket in the Kir6.1/SUR2B (vascular) channel in propeller P1 conformation (PDB ID 7MIT).

K_ATP_ inhibitors such as sulfonylureas and glinides have been used to treat type 2 diabetes for more than half a century. Since the discovery that the gain of function K_ATP_ mutations cause neonatal diabetes (Gloyn et al., 2004; Koster et al., 2000), they have also become the first-line medications for the affected patients (Pipatpolkai et al., 2020). In the initially published cryo-EM structure of the pancreatic K_ATP_ channel in the presence of Glib at a moderate resolution, Li et al. (2017) tentatively assigned cryo-EM density near the interface of L0 and the TM bundle above NBD1 of SUR1 to be Glib (Li et al., 2017), based on previous mutational studies that S1238Y in TM16 of TMD2 and Y230A and W232A in L0 disrupt Glib binding and inhibition (Ashfield et al., 1999; Vila-Carriles et al., 2007). However, later, higher resolution structures revealed Glib cryo-EM density in a pocket located in the TM region above NBD1 framed by TM helices from both TMD1 (TM6, TM7, TM8, and TM11) and TMD2 (TM16 and TM17) and further stabilized by a hairpin loop in L0 (Martin et al., 2017a; Wu et al., 2018; Fig. 7). The binding site was validated by comparing cryo-EM maps of K_ATP_ with or without Glib (Martin et al., 2019; Wu et al., 2018) and by structure-guided mutation-Glib response correlation studies (Martin et al., 2017a).

The Glib binding site offers mechanistic interpretations of previous mutation studies. In the structure, S1238 juxtaposes the cyclohexyl moiety of Glib. Mutation of this residue to a bulkier amino acid would create steric clash, thus explaining why substituting the serine with tyrosine, which is the amino acid at the equivalent position in SUR2, reduces Glib affinity and increases reversibility of Glib inhibition (Ashfield et al., 1999; Yan et al., 2006). Indeed recent structures of a Kir6.1/SUR2B vascular K_ATP_ channel bound to Glib show the drug in the same pocket but with slightly different poses (Sung et al., 2021), likely due to two amino acid variations between the two SUR isoforms (S1238 in SUR1 versus Y1205 in SUR2, and T1242 in SUR1 versus S1209 in SUR2; Fig. 7, A ii and C). This explains why Glib binds with lower affinity to SUR2 and is less potent in inhibiting SUR2 K_ATP_ channels. In contrast to residues directly surrounding the Glib binding pocket, Y230 and W232 located in L0 are too distant to interact with Glib directly. Instead, the L0 amphipathic helix forms a series of interactions with TM helices that line the Glib binding pocket (Fig. 7 A iii). Mutation of these L0 residues would destabilize the binding pocket and indirectly reduce Glib binding and inhibition.

Subsequent studies found that other K_ATP_ inhibitors, including repaglinide, a glinide, as well as carbamazepine, an anticonvulsant best known as a voltage-gated sodium channel blocker but recently shown to also inhibit K_ATP_ channels with high affinity (Chen et al., 2013), share a common binding pocket but engaging distinct sets of residues (Martin et al., 2019). For example, repaglinide interacts with many residues that Glib does but does not make contact with S1238 (Ding et al., 2019; Martin et al., 2019; Fig. 7 B). These observations indicate the pocket is sufficiently flexible to accommodate structurally diverse molecules. Note in channel structures where SUR1 NBDs are dimerized, rearrangements of the TM helices preclude drug binding (Lee et al., 2017). Thus, pharmacological inhibitor binding and NBD dimerization result in two mutually exclusive structural conformations. This is consistent with functional studies showing that the effects of pharmacological inhibitors on channel activity are more pronounced in the presence of MgATP/MgADP (Proks et al., 2013) and that the inhibitors render channels unable to respond to MgATP/MgADP stimulation (Proks et al., 2013; Zhou et al., 2014).

As alluded to earlier, in all cryo-EM structures of K_ATP_ channels formed by separate SUR and Kir6 and bound to pharmacological inhibitors, the distal N-terminus of Kir6 (KNtp) is lodged in the SUR ABC core central cavity next to the bound drug (Martin et al., 2019; Sung et al., 2021; Fig. 4 B; also see Fig. 13, A and B). A similar structural observation was made using an artificial construct of SUR1 ABC transporter core (208–1,582) fused to KNtp from Kir6.1 (1–20) by a 26–amino acid linker bound to Repa (Ding et al., 2019). It has long been recognized that the N-terminus of Kir6 contributes to pharmacological inhibitor binding and gating (Koster et al., 1999a; Kühner et al., 2012). The cryo-EM structures resolve the long-standing puzzle of how KNtp participates in pharmacological inhibitor actions. Furthermore, they show that these inhibitors reduce channel activity not only by preventing NBD dimerization, which prevents MgADP stimulation, but also by trapping KNtp inside the SUR ABC core central cavity, which prevents Kir6 channels from opening, as demonstrated by crosslinking experiments shown in Fig. 4 B.

In addition to inhibiting channel activities, the aforementioned inhibitors also all promote wild-type K_ATP_ channel biogenesis and act as pharmacological chaperones to correct channel folding/assembly defects caused by disease mutations (reviewed in Martin et al., 2020). Deleting the N-terminus of Kir6.2 compromises the ability of these inhibitors to correct disease mutation-induced channel trafficking defects (Devaraneni et al., 2015). Not only does KNtp mediate the effect of pharmacological chaperones but it is also crucial for wild-type channel assembly (Devaraneni et al., 2015; Schwappach et al., 2000). The structural relationship between KNtp, SUR1 ABC core, and pharmacological inhibitors explains the dual function of KNtp in regulating channel assembly and gating both under physiological and pharmacological conditions.

### K_ATP_ openers

**Figure 8.**
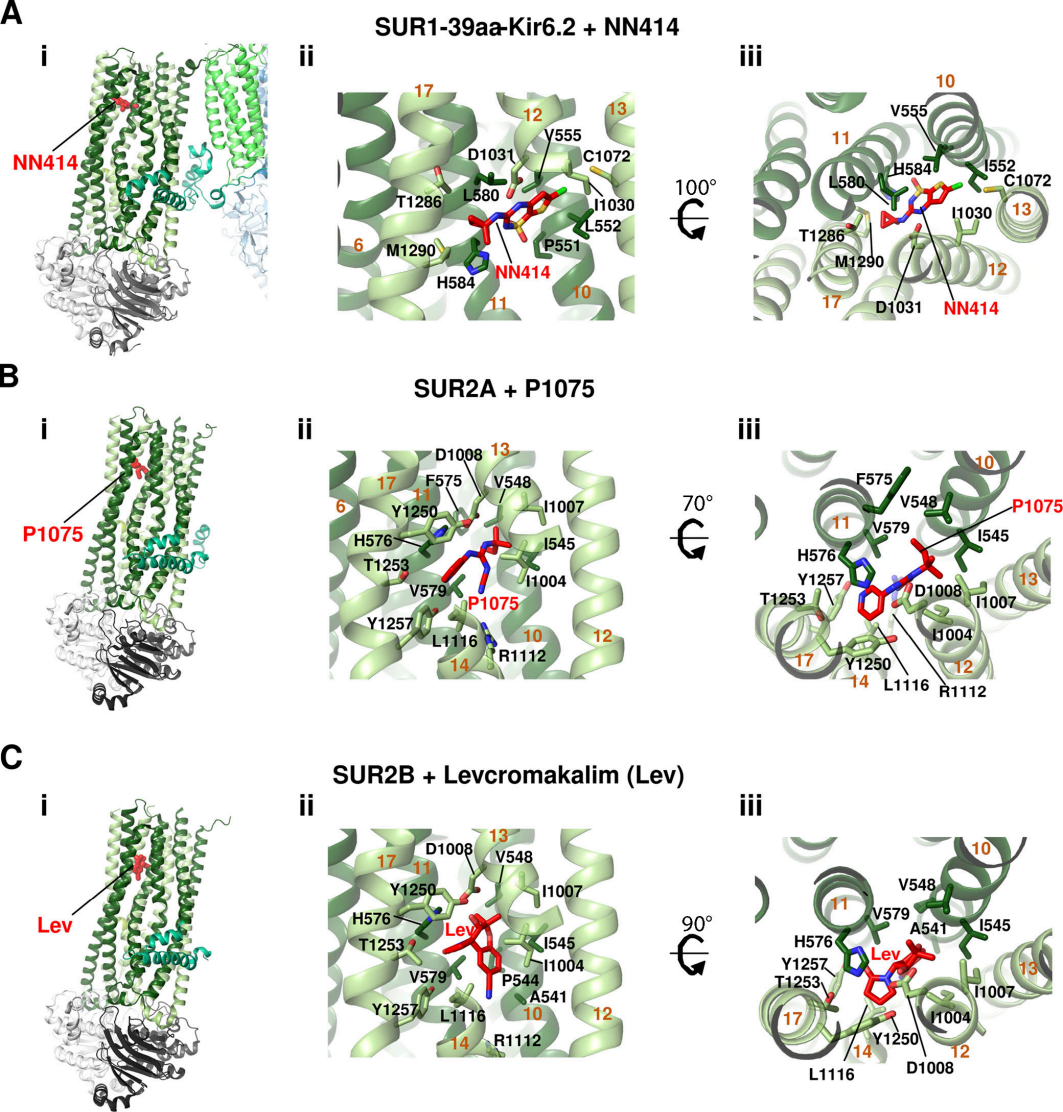
**Binding sites of K**_**ATP**_ **channel openers. (A)** SUR1-39aa-Kir6.2 fusion (pancreatic) K_ATP_ channel bound to SUR1-selective opener NN414 (PDB ID 7W4O). **(A i)** Overview of the NN414 (red carbons) binding pocket from the side. TM helix numbers in this and subsequent panels are labeled in dark orange. **(A ii)** Detailed side view of the NN414 binding pocket with surrounding residues shown as sticks and labeled. **(A iii)** NN414 binding pocket viewed from the top. **(B)** SUR2A bound to SUR2 selective opener P-1075 (PDB ID 7VLU). **(B i)** Overview of P-1075 (red carbons) binding pocket from the side. **(B ii)** Closeup side view. **(B iii)** Close-up top view. **(C)** SUR2B bound to SUR2 selective opener Levcromakalim (Lev; PDB ID 7VLT). **(C i)** Overview of the Lev (red carbons) binding pocket from the side. **(C ii)** Closeup side view. **(C iii)** Close-up top view. Note in B i and C i, TMD0 of SUR2A and SUR2B are not resolved in the cryo-EM maps and are therefore not modeled (Ding et al., 2022).

The binding sites of K_ATP_ channel openers in SUR1, SUR2A, and SUR2B (Fig. 8) were recently revealed in two studies. The first reported cryo-EM structure of SUR1-39aa-Kir6.2^H175K^ fusion channel bound to MgADP and the SUR1 selective opener NN414 (Wang et al., 2022), and the second reported SUR2A or SUR2B alone bound to SUR2-selective openers P1075 or levcromakalim (Ding et al., 2022). NN414 is positioned in the TM region coordinated by residues from TM10 and TM11 of TMD1, and TM12, TM13, and TM17 of TMD2 (Fig. 8 A). The binding site is supported by functional validation experiments. To determine binding sites of SUR2 targeting openers, only SUR2A or 2B proteins were used due to difficulty in obtaining high-resolution reconstructions of the heteromeric SUR2A/2B-Kir6 channels, which likely reflects instability or conformational heterogeneity of the complex. The binding sites for P1075 and levcromakalim are similar, with overlapping but nonidentical interacting residues. They are located in the general TM region where NN414 binds in SUR1, but the pocket is lined by TM10, TM11, TM12, TM14, and TM17 instead (Fig. 8, B and C). Two residues H576 and T1253 are deemed essential for P1075 binding as mutation of either residue to alanine nearly eliminated channel response to the opener. Comparison of SUR2 and SUR1 sequences suggests that I1004 and T1253, which are Leu and Met, respectively, in SUR1, account for the isoform specificity of P1075. The detailed binding site models derived from the cryo-EM structures of the different SUR isoforms bound to different isoform selective openers thus offer insights into the affinity and specificity of these compounds.

In all opener bound structures, the SUR NBDs are dimerized with the degenerate NBD1 occupied by MgATP and the consensus NBD2 by MgADP. It is known that the effect of potassium channel openers is dependent on Mg–nucleotides, and the channel openers in turn enhance Mg–nucleotide stimulation (reviewed in Mannhold, 2004). From the structures it appears that dimerization of the NBDs brings the SUR ABC module TMDs into a conformation that favors potassium channel opener binding, and binding of the openers in turn further stabilizes SUR in an NBD dimerized conformation to facilitate channel activation.

## Structural dynamics of K_ATP_ channels

In single-particle cryo-EM, multiple conformations of a given protein sample may be captured, which may correspond to different functional or transitional states (Nogales and Scheres, 2015; Sigworth, 2007). In all K_ATP_ channel structures resolved to date, the Kir6.x tetramer TM region and the surrounding TMD0 of SUR have the highest resolution, indicating it is the most stable part of the channel structure. Heterogeneity in the cytoplasmic domain (CTD) of Kir6.2 and the ABC core of SUR is commonly observed, and in the case of the Kir6.2-CTD, conformational dynamics have been reported to be ligand-dependent (Sung et al., 2022). Linkers that connect major domains of the SUR protein, as well as the N- and C-terminal 30–40 amino acids of the Kir6 subunit are generally poorly resolved in the cryo-EM maps, indicating they are highly dynamic. Interestingly, some of these disordered regions become more structured in the presence of specific ligands or in a particular conformation to reveal how these dynamic parts of the channel may regulate channel function. Moreover, by correlating different conformations with functional states of the channel, the structural pathways that the channel undertakes to transition between different functional states have begun to emerge, as we discuss below.

### Conformation dynamics of Kir6

While the Kir6.2 TMD is relatively stable, the CTD of Kir6.2 is dynamic. Wu et al. (2018) first identified two distinct Kir6.2-CTD conformations, which they call T- (for tense) or R- (for relaxed) states, in SUR1-39aa-Kir6.2 fusion channels (Wu et al., 2018). The Kir6.2-CTD in the T-state is rotated clockwise by ∼10.6–12.5° (viewed from the extracellular side perpendicular to the membrane) and closer to the lipid bilayer by 3–4.2 Å compared with the R-state. Both states were observed in channels imaged in three different ligand conditions: with ATPγS alone, ATPγS plus Glib, or with MgADP plus NN414.

**Figure 9.**
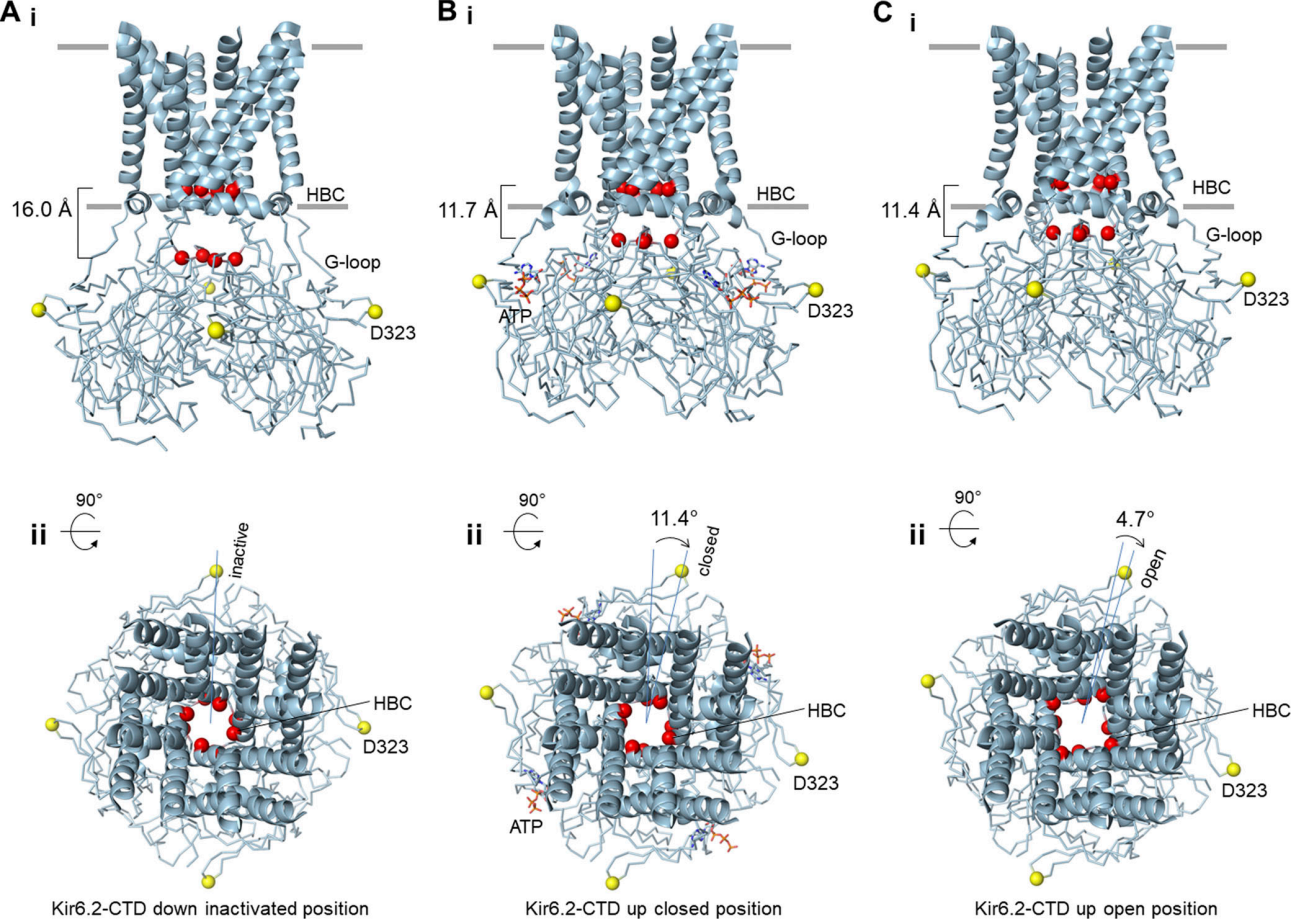
**Kir6.2 CTD conformations. (A)** Cryo-EM structure of unliganded (apo) Kir6.2 with CTD in the down conformation corresponding to an inactive channel (PDB ID 7UQR). **(B)** Cryo-EM structure of ATP-bound Kir6.2 in CTD-up conformation corresponding to an inhibited channel (PDB ID 7TYS). **(C)** Cryo-EM structure of open Kir6.2^G334D, C166S^ in the CTD-up open conformation (PDB ID 7S5T). All three structures are shown from the side (i) and the top (ii). F168 at the helix bundle crossing (HBC) gate and G295 at the G-loop gate are shown as red spheres and the distance between the HBC and G-loop gate is shown on the left of the side view. In the top-down view, D323 in the CTD is used to measure the degree of rotation between the three conformations.

**Figure 10.**
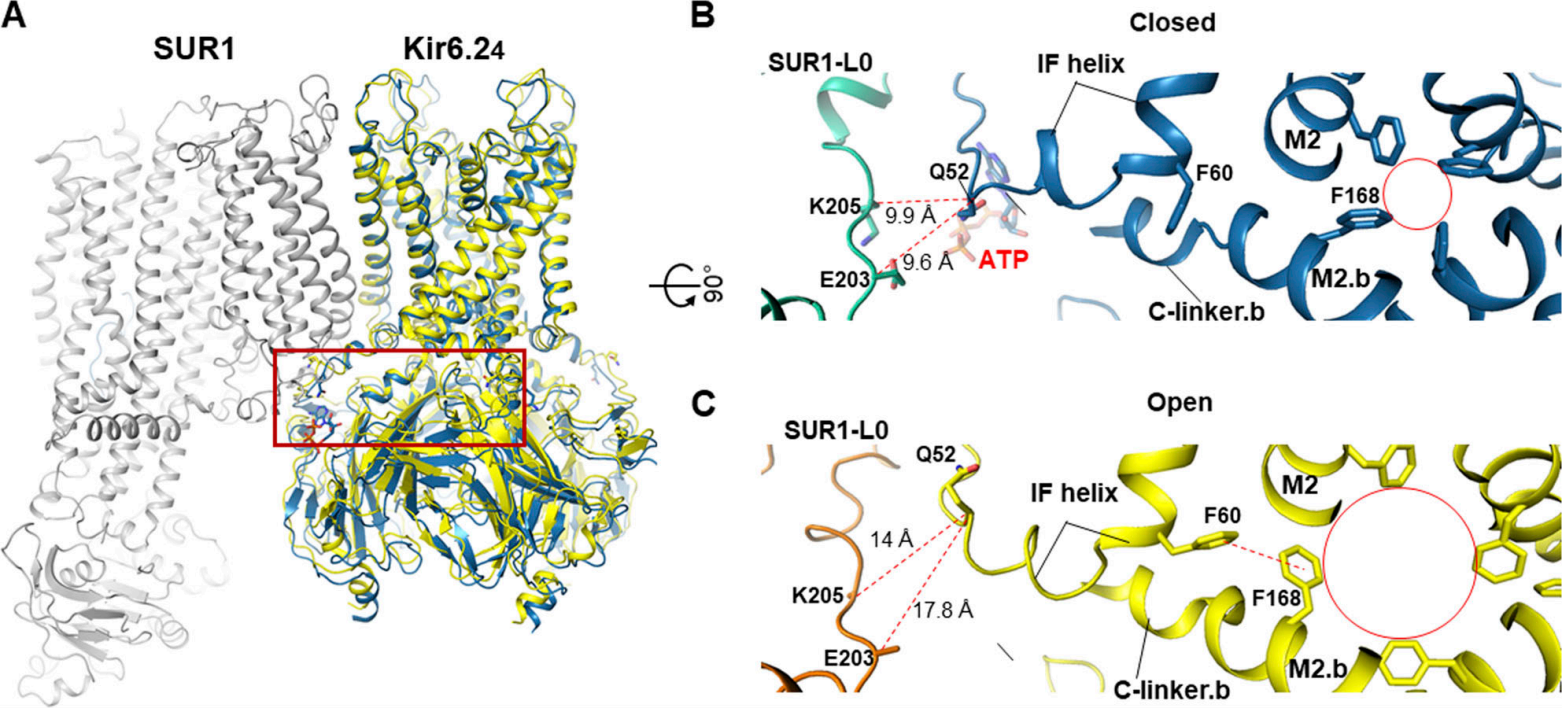
**Remodeling of the cytoplasmic SUR1-Kir6.2 interface from ATP-inhibited to open conformation. (A)** Overlay of the Repa/ATP bound closed K_ATP_ structure in CTD-up conformation (PBD ID 7TYS; blue) and the open Kir6.2^G334D, C166S^ structure (PDB ID 7S5X; yellow). The red box indicates regions shown in closeup, top-down view in B and C. **(B and C)** Comparison of key molecular differences between ATP-bound closed conformation (B) and SUR1 NBD-dimerized, Kir6.22^G334D, C166S^ open conformation (C). Note the reorientation of the F60 and F168 side chains and the enlargement of the helix bundle crossing gate in the open conformation (red circle). The distances between Q52 of Kir6.2 and E203 or K205 of SUR1-L0 (red dashed lines) are also significantly increased in the open conformation, which disfavors ATP binding.

**Figure 11.**
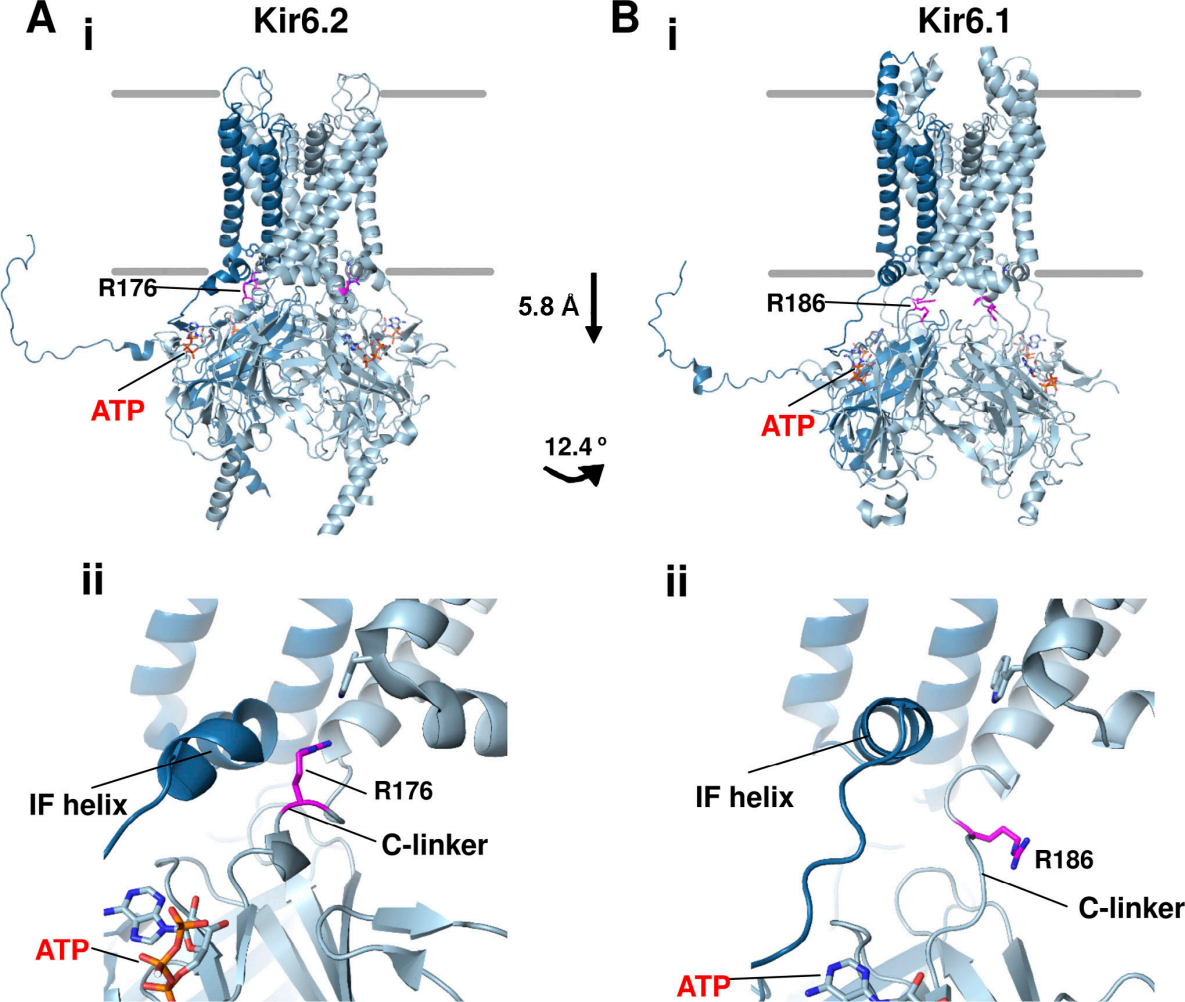
**Comparison of ATP-bound Kir6.1 and Kir6.2 structures. (A i)** Overall structure of Kir6.2 bound to ATP (red label) viewed from the side showing CTD in the up position and R176 (magenta carbons) close to the plasma membrane (PDB ID 7TYS). **(A ii)** Closeup view of the structure near the inner membrane. **(B)** Same as A but of the structure of Kir6.1 bound to ATP (PDB ID 7MIT). Note the Kir6.1-CTD is translated toward the cytoplasm by 5.8 Å and counterclockwise rotated by 12.4° (viewed from the top). The interfacial helix (IF helix) straightens into a full helix and the C-linker unwinds into a loop, and R186 in the C-linker is too removed from the inner membrane to interact with activating phospholipids in the predicted PIP_2_ binding pocket.

Another recent study by Sung et al. (2022) analyzing five different cryo-EM datasets of channels formed by the coexpression of SUR1 and Kir6.2 collected alternatively in Glib plus ATP, Repa plus ATP, carbamazepine, ATP alone, or no ligand (apo) also reported two Kir6.2 CTD conformations (Sung et al., 2022). The two conformations, which are referred to as CTD-up and CTD-down, differ in rotation and distance to the plasma membrane (Fig. 9, A and B), and are qualitatively similar to the T- and R-states, respectively. In this latter study, however, the distribution of particles in the two conformations shows ligand-dependency. In channels bound to ATP and the high-affinity inhibitor Glib and Repa, the majority of the particles have the CTD in the up conformation, channels bound to the lower affinity inhibitor carbamazepine or ATP alone are predominantly in the CTD-down conformation, and in the apo condition, all particles have the CTD in the down conformation. Molecular analysis of the two conformations suggests that pharmacological inhibitors and ATP stabilize a network of interactions that bind key structural elements in SUR1 and Kir6.2 together, including intracellular loops of the TMD0+L0 of SUR1 and the interfacial helix and the C-linker of Kir6.2 to favor the CTD up conformation (see Fig. 10). Note, in the CTD-down conformation, the C-linker unwinds into a loop such that positively charged residues, previously shown to be critical for channel interaction with PIP_2_, cannot reach PIP_2_ to support channel opening (see Fig. 11).

What are the functional correlates of the two Kir6.2 CTD conformations? A leading hypothesis is that the CTD-down conformation is inactive, while the CTD-up conformation enables ligand modulation. Previous studies have found several mutations at the Kir6.2–Kir6.2 or SUR1–Kir6.2 subunit interface can cause rapid, spontaneous decay of K_ATP_ currents, referred to as inactivation (Borschel et al., 2017; Lin et al., 2008; Lin et al., 2003; Shyng et al., 2000b). Inactivation can be prevented or reversed by exposing channels to PIP_2_. Inactivation can also be recovered by exposing channels to ATP in a concentration- and time-dependent manner. It has been proposed that inactivation mutations lead channels into a conformation that is unable to interact with PIP_2_ (Borschel et al., 2017; Lin et al., 2003). Exposure to ATP shifts the channel back to a conformation conducive to PIP_2_ interaction such that channels can open briefly again before sinking back to the inactivated conformation. PIP_2_ can also prevent or reverse inactivation by shifting the equilibrium toward the PIP_2_-bound open state. The structural characteristics of the Kir6.2 CTD-down conformation are consistent with a channel unable to interact with PIP_2_ and open, and this conformation therefore likely corresponds to the inactivated state (Borschel et al., 2017; Lin et al., 2003). ATP, by acting as a molecular glue at the key subunit interfaces, shifts the equilibrium of the inactivation mutant Kir6.2 CTD toward the up conformation such that upon ATP removal, the CTD is primed to interact with PIP_2_ for opening.

How does the channel transition from ATP-bound closed state to the open state? Attempts to capture wild-type Kir6.2 in its open state by adding diC8 PIP_2_ and activating SUR1 with MgATP/MgADP and potassium channel openers have so far been unsuccessful. However, employing a mutant Kir6.2 that carries a mutation G334D, which prevents ATP binding, and a C166S mutation in the inner helix of Kir6.2 known to stabilize the channel in the open state, Zhao and MacKinnon (2021) recently reported the first open K_ATP_ channel structure. In this study, channels were formed by coexpressing SUR1 and mutant Kir6.2, and MgATP was added to induce SUR1 NBD dimerization. As expected, despite high concentrations of MgATP, no ATP was bound at Kir6.2 due to the G334D mutation. The radius at the bundle crossing gate near F168 is ∼3.3 Å, significantly wider than that seen in the closed WT channel (∼1 Å), and expected to allow K^+^ conduction (Fig. 9 C; and Fig. 10, A and C). Compared with the ATP bound closed CTD-up conformation, the CTD is further clockwise rotated (extracellular view) by ∼5°, with significant structural rearrangements at the interfacial helix and the C-linker (Fig. 10, B and C). Most notably, F60 on the interfacial helix is positioned to stabilize the side chain of F168. The ATP binding site also widens such that ATP can no longer fit snugly. A subsequent study by Wang et al. (2022) on a mutant fusion channel SUR1-39aa-Kir6.2^H175K^, in which the Kir6.2 H175K mutation was introduced to enhance PIP_2_ binding, reached similar conclusions (Wang et al., 2022). In this study, two structures were derived from the same sample imaged in the presence of MgADP, NN414, and diC8-PIP_2_: a closed Kir6.2 bound to ADP and a pre-open Kir6.2 devoid of ADP. In both structures, the Kir6.2 CTD is docked close to the membrane, but the CTD of the pre-open structure is further rotated by ∼6° clockwise relative to the closed structure from the extracellular view. There is an expansion of the cytoplasmic end of the inner helix to widen the helix bundle crossing gate to 3 Å, which is only slightly narrower than the Kir6.2^G334D, C166S^ structure. In the pre-open structure, the side chain of Kir6.2-Q52, which faces the nucleotide-binding pocket in the closed structure, is flipped to face the intracellular loops of the SUR1 TMD0, while the side chain of E51 is flipped to the opposite direction to occupy the nucleotide-binding pocket. These studies give support to the notion that the open channel conformation is not compatible with ATP/ADP binding at Kir6.2 and that ATP/ADP inhibits the channel by stabilizing the channel in the closed conformation (Enkvetchakul et al., 2000).

With the different conformations reported, we can now envision a structural pathway for Kir6.2 to transition between three distinct functional states: an inactivated state, where the CTD is corkscrewed down and untethered from the membrane, a closed state, where the CTD is bound to ATP/ADP and corkscrewed up to be close to the membrane, and an open state, where the CTD is further rotated and the inner helix splayed open near the helix bundle crossing (Sung et al., 2022; Fig. 9). This functional interpretation further provides an explanation for a long-known difference in isoform-specific channel opening probabilities. Consistent observations have been reported that among channels assembled from different SUR and Kir6 isoforms, Kir6.1-containing channels all have very low open probability compared with Kir6.2-containing channels, regardless of which SUR is their partner (Kondo et al., 1998; Satoh et al., 1998). In the vascular Kir6.1/SUR2B structure bound to Glib and ATP, the Kir6.1 CTD is seen only in the corkscrewed down conformation (Fig. 11 A), where the Kir6.2-R176 equivalent residue Kir6.1-R186, thought to be critical for PIP_2_ binding, is far away from the membrane for PIP_2_ interaction (Sung et al., 2021; Fig. 11 B). This structural difference may explain the low open probabilities of Kir6.1-containing K_ATP_ channels (Yamada et al., 1997). Why the Kir6.1 CTD adopts a conformation different from Kir6.2 under the same ligand condition and what allows for the transition of the Kir6.1 CTD to the up conformation, which presumably is required for channel opening, remain open questions.

### Conformational dynamics of SUR

**Figure 12.**
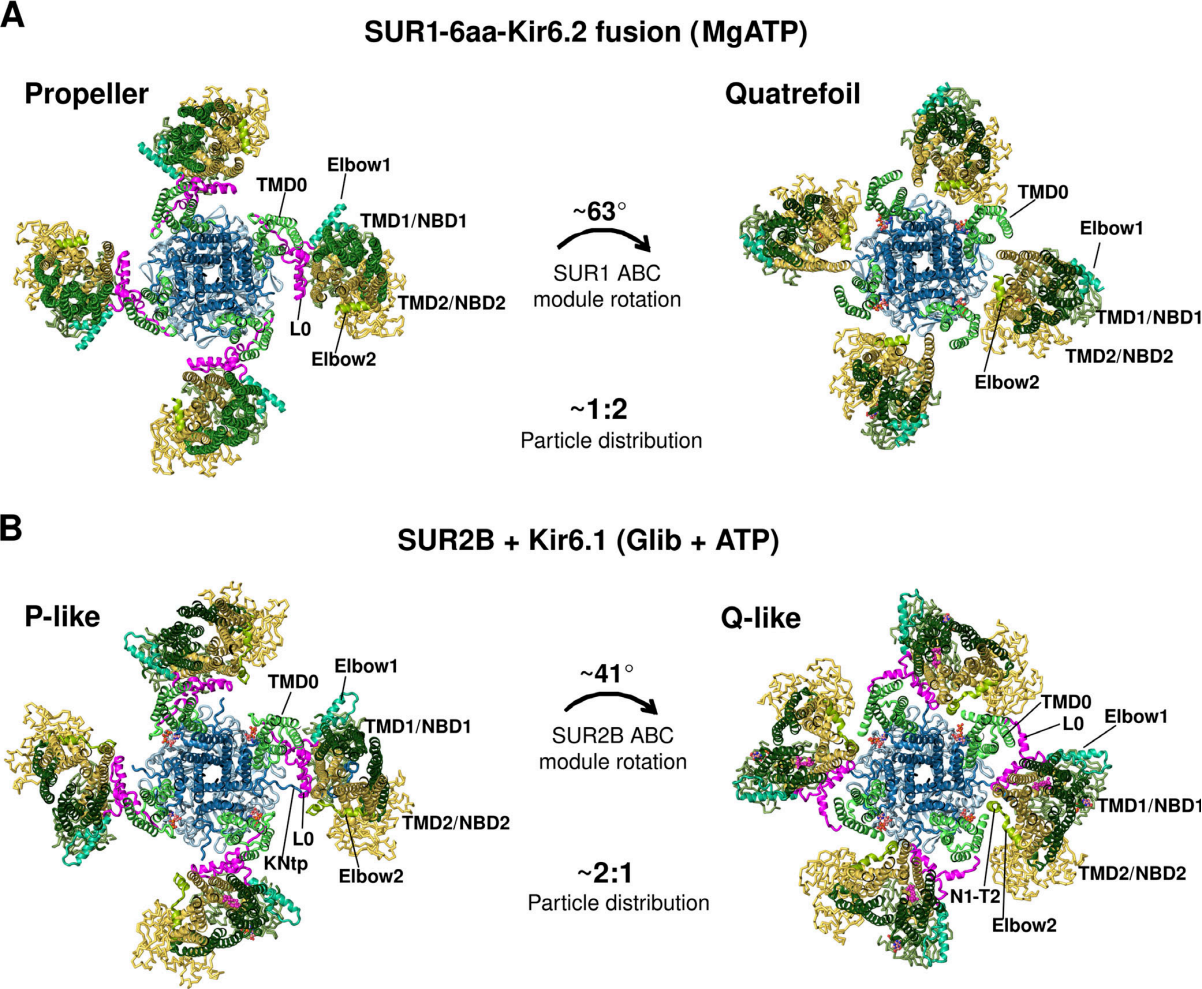
**SUR conformational dynamics. (A)** Human SUR1-6aa-Kir6.1 fusion channel structure bound to MgATP/MgADP at SUR1 NBD1/NBD2 and ATP at Kir6.2 in propeller (PDB ID 6C3P), and quatrefoil conformation (PDB ID 6C3O). The ratio of the propeller versus quatrefoil conformation was ∼1:2. In both propeller and quatrefoil conformations, SUR1 NBDs were dimerized. SUR1-L0 cryo-EM density was not observed in the quatrefoil conformation and therefore missing in the model (Lee et al., 2017). **(B)** Rat SUR2B+Kir6.1 vascular K_ATP_ bound to Glib and ATP in propeller (P)-like (PDB ID 7MIT) and quatrefoil (Q)-like conformation (PDB ID 7MJO). The ratio of the propeller versus quatrefoil conformation was ∼2:1. In both P-like and Q-like conformations, SUR2B NBDs were separate. SUR2B-L0 cryo-EM density was well-resolved and SUR2B-L0 was modeled in both conformations (Sung et al., 2021).

The SUR subunit, in particular its ABC module, is much more dynamic than Kir6.2 in all cryo-EM structures. As mentioned before, multiple SUR conformations have been observed, although the physiological relevance and functional significance of these conformational alternatives are unclear. Initial pancreatic K_ATP_ structures bound to Glib with or without ATP show a propeller-like conformation. A subsequent study of a human SUR1-6aa-Kir6.2 fusion channel solubilized in amphipol and imaged in the presence of MgATP revealed two conformations, a dominant quatrefoil conformation and a less populated propeller conformation (Fig. 12 A). However, a recent study from the same group using channels formed by coexpressing SUR1 and Kir6.2^G334D, C166S^ (solubilized in the detergent glycol diosgenin [GDN], but imaged similarly in the presence of MgATP, with SUR1 NBDs dimerized) did not capture the quatrefoil conformation, although the SUR1 subunit adopts a heterogeneous range of clockwise rotations (five subclasses) around the Kir6.2 central axis (Zhao and MacKinnon, 2021). Whether the prominent difference in SUR1 conformations between the two studies reflects a difference in the constructs used (i.e., fusion versus SUR1/Kir6.2 coexpression), experimental conditions, or data processing is unclear. In contrast, all SUR1 NBD-dimerized K_ATP_ structures published by the Chen group using SUR1-39aa-Kir6.2 fusion constructs are in the propeller conformation (Wang et al., 2022; Wu et al., 2018). Comparison of the structure of a SUR1-39aa-Kir6.2^H175K^ fusion channel in the closed and pre-open conformations revealed a significant difference in the cytoplasmic half of the TMD0 domain (Wang et al., 2022). Specifically, the cytoplasmic half of TMD0 moves outward as the Kir6.2 channel opens. The ICL1 of TMD0 becomes more ordered and interacts with the N-terminal β strand and interfacial helix of Kir6.2. Likewise, L0 also moves outward such that K205 is too distant to coordinate ATP/ADP binding in Kir6.2, which favors ATP/ADP dissociation and channel opening (similar to what is shown in Fig. 10 C).

The structures discussed so far suggest that propeller conformation might be the norm. However, adding more pieces to the puzzle, a recent study of the vascular Kir6.1/SUR2B structure bound to Glib and ATP presented four conformations, two resembling a propeller and two a quatrefoil (Sung et al., 2021; Fig. 12 B). The ratio of the particles in the propeller versus the quatrefoil conformations is ∼2:1. In all conformations, the SUR2B ABC module has an inward-facing structure with the two NBDs clearly separated, and only subtle differences are observed between the two propeller subclasses and between the two quatrefoil subclasses. In stark contrast to the NBD-dimerized human pancreatic fusion K_ATP_ quatrefoil structure (Lee et al., 2017), the L0 in the vascular quatrefoil-like structure is well resolved (compare Fig. 12, A and B). The large rotation of the ABC module in the quatrefoil-like conformations markedly reduces the contact surface area with TMD0.

**Figure 13.**
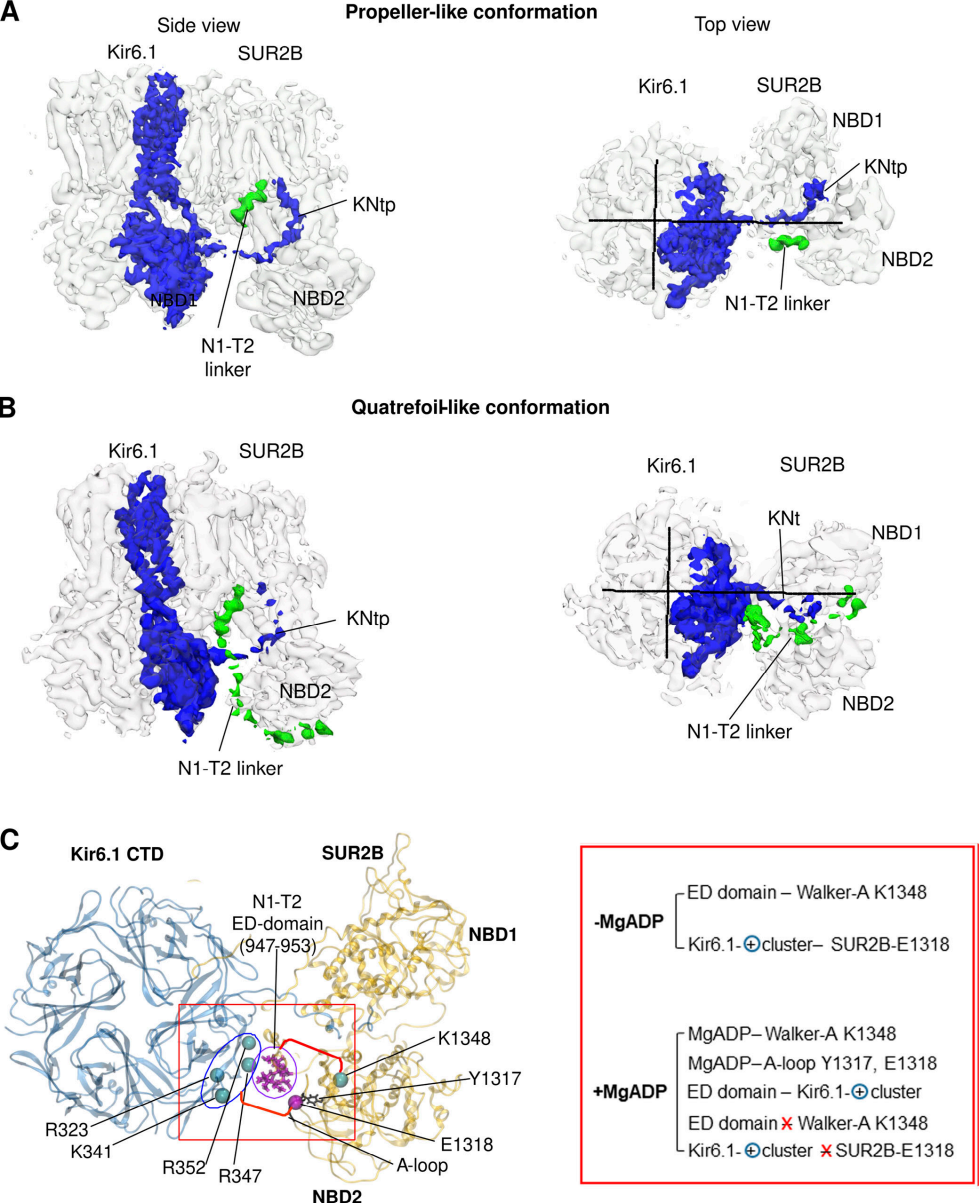
**The NBD1–TMD2 (N1–T2) linker revealed in the quatrefoil-like conformation of Glib/ATP bound Kir6.1/SUR2B vascular K**_**ATP**_ **channels. (A)** Cryo-EM map of (Kir6.1)_4_-SUR2B in propeller-like conformation (EMD-23864, filtered to 5 Å and contoured to 0.7 σ) with the density of one Kir6.1 subunit and the SUR2B N1–T2 linker shown in blue and green, respectively, in side and top views. **(B)** Same as A but in quatrefoil-like conformation (EMD-23880, filtered to 5 Å, and contoured to 0.5 σ). **(C)** Bottom view of the (Kir6.1)_4_-SUR2B structure in the quatrefoil-like conformation (PDB ID 7MJO) showing the spatial relationship between the second half (purple sticks) of the ED domain (^947^EDEDEEEEEEEDEDD^961^) in the N1–T2 linker, the Kir6.1 positively charged residue cluster (blue spheres), and residues in SUR2B-NBD2 including K1348 of the Walker A motif (blue sphere), and Y1317 (blue sticks) and E1318 (purple sphere) in the A-loop, involved in Mg-nucleotide binding. The red box on the right shows dominant interactions observed during three independent 1-μs MD simulations in the absence of MgADP and which are disrupted in the presence of MgADP (red X) due to competition of the NBD2 residues for MgADP binding. The blue-circled + denotes Kir6.1 positively charged residue cluster. A and B are adapted from Fig. 5 A, and C from Fig. 6 B in Sung et al. (2021).

Another significant finding from the vascular K_ATP_ quatrefoil-like conformation is that the cryo-EM density of a previously elusive linker between NBD1 and TMD2 (N1–T2 linker) becomes visible and is sandwiched between the SUR2B NBD2 and the cytoplasmic domain of Kir6.1 (Fig. 13). This location differs from that of the corresponding linker in phosphorylated CFTR (Zhang et al., 2018) or the yeast cadmium transporter Ycf1p (Bickers et al., 2021). In CFTR and Ycf1p, the N1–T2 linker lies at the outer surface of NBD1 instead. MD simulations probing the interactions between SUR2B-NBD2, the N1–T2 linker, and Kir6.1-CTD showed that a stretch of 15 consecutive negatively charged amino acids in the N1–T2 linker, called the ED domain (Karger et al., 2008), preferably interacts with Walker A residue K1348 in NBD2 in the absence of MgADP at NBD2, while at the same time, a negatively charged residue E1318 in the A-loop of NBD2 interacts with a cluster of positively charged residues in the Kir6.1-CTD (Sung et al., 2021; Fig. 13 C). However, when NBD2 is occupied by MgADP, the Walker A K1348 and E1318 in the A-loop are engaged in MgADP binding instead, which breaks NBD2 free from the ED domain and positively charged residues in Kir6.1 (Sung et al., 2021; Fig. 13 C). This would allow NBD2 the freedom to dimerize with NBD1. These observations suggest that the ED domain serves as an autoinhibitory motif to prevent spontaneous NBD dimerization in the absence of MgADP. The physiological relevance and significance of the structural findings are supported by previous biochemical and electrophysiological data indicating that SUR2-E1318 forms a salt bridge with R347 in Kir6.1 and that disruption of this interaction facilitates channel activation by MgADP and the potassium channel opener pinacidil while reducing Glib inhibition (Lodwick et al., 2014), gating behaviors that implicate enhanced NBD dimerization. Taken together, these findings highlight the need for more studies to understand the structural and functional dynamics of SUR in the K_ATP_ complex.

## Conclusion and outlook

The past 5 yr have been an exciting time for the K_ATP_ channel structure–function field. There are finally high-resolution 3-D structures of the channels or channel subunits with which to correlate decades of biochemical and functional data. Despite the tremendous progress, major knowledge gaps remain.

First, what are the structural and functional roles of intrinsically disordered regions that have yet to be visualized by cryo-EM? Structural regions like the KNtp and SUR N1–T2 linker were poorly resolved in most cryo-EM structures but became much more ordered under certain experimental conditions, such as in the presence of pharmacological inhibitors and specific K_ATP_ isoforms. There remain a number of disordered regions including the C-terminus of Kir6 and the linker connecting the TMD1 and NBD1 in the SUR ABC module that awaits to be resolved. Future work using different conditions to stabilize these regions and comparing and contrasting different K_ATP_ isoforms will hopefully offer new insights. Second, what is the structural basis that underlies the different gating properties seen in different K_ATP_ isoforms? Recent structures of the Glib/ATP bound vascular K_ATP_ channels offer insights into why Kir6.1-containing channels have a low spontaneous open probability. However, we still do not know the basis of the different ATP and MgATP/ADP sensitivities in pancreatic, cardiac, and vascular K_ATP_ channels. A direct comparison of all three major channel isoforms under the same ligand conditions will be needed to address this question. These comparisons may also reveal novel mechanisms of channel regulation as illustrated by the analysis of the N1–T2 linker in the SUR2B quatrefoil-like conformation. Third, does PIP_2_ bind to the predicted PIP_2_ binding pocket for physiological gating of K_ATP_ channels? Fourth, does the quatrefoil conformation represent a physiological state? Lastly, how do disease mutations disrupt channel biogenesis, trafficking, and gating? While the structures have improved our understanding of how mutations of certain residues such as those located at the SUR1–Kir6.2 transmembrane contact sites disrupt channel trafficking to the cell surface or how mutations of residues coordinating ATP binding reduce ATP sensitivity, the structural mechanisms by which many mutations not directly impacting ligand binding affect channel function are still poorly understood and will require structures of mutant channels for answers. Such structures will ultimately help map out the conformation pathways through which the various gating events occur.

The structural knowledge we now have also presents new opportunities for structure-based drug development to transform the current K_ATP_ pharmacology landscape. Despite the rich history and clinical use of K_ATP_ channel modulators, major challenges remain due to the lack of isoform-specific K_ATP_ modulators and a large number of disease mutations that do not respond to currently available drugs. The structures bound to inhibitors and activators suggest these pockets can accommodate chemically diverse compounds, which could be exploited to improve isoform specificity and other properties, such as permeability through the blood-brain barrier, for specific disease applications such as DEND syndrome. Besides small molecule inhibitors, a family of protein toxins isolated from centipede venom bind directly to human Kir6.2 to inhibit channel activity (Ramu and Lu, 2019; Ramu et al., 2018). The most potent one, SpTx1, inhibits human K_ATP_ with a Kd of 15 nM, and in a mouse expressing human Kir6.2, the toxin was shown to enhance glucose-stimulated insulin secretion without triggering insulin secretion at basal glucose (Ramu et al., 2022), suggesting that the toxin may preferentially bind closed channels. These studies highlight the potential of novel chemical spaces for developing new therapeutics.

Beyond structures of purified SUR/Kir6 channel complexes, expansion of K_ATP_ channel structure biology to include other interacting proteins, including COPI, 14-3-3 proteins, syntaxin 1 and ankyrin, to name a few, will help us understand how these proteins regulate channel trafficking and function. Finally, by combining single particle cryo-EM and cryo-electron tomography, one sees a future where the organization and dynamics of K_ATP_ channel signaling complexes can be visualized in situ, and mechanisms that lead to the breakdown in normal channel trafficking and function can be elucidated.
